# Burden analysis of diabetic nephropathy caused by excessive intake of sugar-sweetened beverages in high and low SDI regions

**DOI:** 10.3389/fpubh.2025.1598278

**Published:** 2025-06-27

**Authors:** Xue Wang, Guangyan Cai, Qing Ouyang, Xiangmei Chen

**Affiliations:** State Key Laboratory of Kidney Diseases, Beijing Key Laboratory of Medical Devices and Integrated Traditional Chinese and Western Drug Development for Severe Kidney Diseases, Beijing Key Laboratory of Digital Intelligent TCM for the Prevention and Treatment of Pan-vascular Diseases, Department of Nephrology, First Medical Center of Chinese PLA General Hospital, National Clinical Research Center for Kidney Diseases, Key Disciplines of National Administration of Traditional Chinese Medicine (zyyzdxk-2023310), Beijing, China

**Keywords:** diabetic nephropathy, excessive intake of sugar-sweetened beverages, socio-demographic index, deaths, DALYs, YLDs, YLLs, ARIMA

## Abstract

**Objective:**

The Global Burden of Disease Study (GBD) 2019 reveals an increasing prevalence of diabetic nephropathy caused by excessive intake of sugar-sweetened beverages in high and low SDI regions from 1990 to 2021.

**Methods:**

This study comprehensively analyzed the burden of DN caused by SSBs in high and low SDI regions from 1990 to 2021 and projected the trends until 2040 using ARIMA models.

**Results:**

The results revealed distinct patterns and trends in age-standardized rates of deaths, DALYs, YLDs, and YLLs between the two regions. In high SDI regions, the age-standardized rates of deaths, DALYs, YLDs, and YLLs exhibited a consistent upward trend from 1990 (0.044, 1.425, 0.537, 0.888 per 100,000) to 2021 (0.096, 2.284, 0.67, 2.154 per 100,000). Projections for the age-standardized rates in high SDI regions from 2021 to 2040 indicate that these upward trends will persist, with deaths, DALYs, YLDs, and YLLs further increasing to 0.127, 3.629, 1.070, and 2.861 per 100,000. Conversely, low SDI regions displayed different trends. Although the age-standardized rates of deaths, DALYs, YLDs, and YLLs also increased from 1990 (0.018, 0.488, 0.071, 0.417 per 100,000) to 2021 (0.020, 0.522, 0.081, 0.441 per 100,000), the rate of increase was relatively slower compared to high SDI regions. Projections for the age-standardized rates in low SDI regions from 2021 to 2040 suggest that these upward trends will continue, with deaths, DALYs, YLDs, and YLLs further increasing to 0.016, 0.498, 0.105, and 0.398 per 100,000.

**Conclusion:**

This study provides a comprehensive analysis of the burden of type 2 diabetes nephropathy caused by excessive intake of sugar-sweetened beverages in high and low SDI regions. The results revealed distinct patterns and trends in age-standardized rates of deaths, DALYs, YLDs, and YLLs between the two regions.

## 1 Introduction

Globally, the excessive intake of sugar-sweetened beverages (SSBs) has been on the rise, emerging as a significant public health concern. SSBs, which include sodas, energy drinks, and fruit-flavored drinks sweetened with added sugars, have been linked to a host of health problems (1, 2). Among these, their contribution to the development of type 2 diabetes mellitus (T2DM) is well-established. Excessive intake of SSBs leads to weight gain, insulin resistance, and impaired glucose tolerance, all of which are key factors in the pathogenesis of T2DM (3, 4). Diabetic nephropathy (DN), a serious complication of T2DM, is a leading cause of end-stage renal disease (ESRD) worldwide (5). It not only impairs the quality of life of patients but also imposes a heavy economic burden on healthcare systems. The development and progression of DN are influenced by a variety of factors, including genetic predisposition, duration and glycemic control of diabetes, hypertension, and dyslipidemia (6–8). In recent years, there has been growing evidence suggesting that dietary factors, particularly the excessive intake of SSBs, may also play a role in the development and progression of DN (9, 10). The results of the National Health and Nutrition Examination Survey indicate that sugary soft drink consumption is associated with albuminuria (11). Excessive intake of SSBs has been shown to induce rapid spikes in blood glucose and insulin levels from high levels of sugars or high fructose cornsyrup, which in conjunction with the large volumes consumed contribute to a high dietary glycemic load (GL) (12, 13). Fructose has also been shown to promote accumulation of visceral adiposity or ectopic fat deposition (14). Visceral adiposity, rather than BMI per se, appears to be the most significant predictor of the risk factors comprising metabolic syndrome, and a growing body of literature shows that metabolic syndrome is a risk factor for CKD (15, 16). Besides, High-fructose corn syrup-sweetened soft drink consumption increases vascular resistance in the kidneys at rest and during sympathetic activation (17).

The Global Burden of Disease (GBD) study is a comprehensive and systematic effort to quantify the health-related loss due to diseases and risk factors worldwide (18, 19). It provides a unique opportunity to assess the global burden of DN caused by excessive intake of SSBs. By analyzing the GBD database, we can obtain detailed information on the incidence, prevalence, deaths, and disability-adjusted life years (DALYs) attributable to DN due to excessive intake of SSBs in different regions and populations. Diabetic nephropathy is exacerbated by excessive intake of SSBs as a complication of diabetes. The Global Burden of Disease (GBD) Study was initiated in the 1990s for the purpose of assessing health outcomes in a timely, valid, and relevant manner (20–22). This study aims to provide a comprehensive analysis of the global burden of DN caused by excessive intake of SSBs using the GBD database. We will examine the trends in incidence, prevalence, deaths, and DALYs of DN due to excessive intake of SSBs over time and across different regions and age-groups. The results of this study will have important implications for public health policy-making and interventions aimed at reducing the burden of DN caused by excessive intake of SSBs. It will also provide valuable insights for further research on the mechanisms underlying the association between excessive intake of SSBs and DN. The results highlight the significant and increasing public health challenge posed by this condition, particularly in high SDI (Socio-demographic Index) regions.

## 2 Materials and methods types

### 2.1 Study data

GBD 2021 represents a multinational collaborative integrated surveillance system, providing comprehensive and systematic estimates of 369 diseases and injuries as well as 87 risk factors from 1990 to 2021 (23–25). We utilized the GBD Results Tool to retrieve data, selecting “Cause of death or injury” under the GBD Estimate directory; “Deaths,” “DALYs,” “YLDs (Years Lived with Disability),” “YLLs (Years of Life Lost)” under the Measure directory; “Number” and “Rate” under the Metric directory; “Excessive intake of sugar-sweetened beverages” under the Cause directory; “All countries and territories” under the Location directory; “1–95 + years” and “ALL-age standardized” under the Age directory; “Both,” “Female,” and “Male” under the Sex directory; and “1990–2021″ under the Year directory. For this study, we acquired estimates of deaths, DALYs, YLDs, and YLLs attributed to DN due to excessive intake of SSBs based on high and low SDI, age, location, and sex from the Global Health Data Exchange query tool (accessed on 14 January 2025)^1^ (26). Subsequently, the retrieved raw data was exported. It is important to note that the GBD initiative is guided by ethical principles governing data collection and utilization, and the study protocol received approval from the Institutional Review Board at the University of Washington (Seattle, WA, United States).

The SDI serves as a metric gaging per capita income, educational attainment, and total fertility rate in each state, with a higher index signifying improved socio-demographic development (27, 28). In GBD 2021, the SDI was employed as a metric, encompassing aspects such as income per capita distribution, average years of education for individuals aged 15 and above, and fertility rates in females under 25, for each location and year under consideration. Categorization of countries was based on their SDI values in 2019, grouped into five categories: low SDI (<0.455), low-middle SDI (≥0.455 and < 0.608), middle SDI (≥0.608 and < 0.690), high-middle SDI (≥0.690 and < 0.805), and high SDI (≥0.805).

### 2.2 Statistical analysis

To measure trends related to deaths, Disability-Adjusted Life Years (DALYs), Years Lived with Disability (YLDs), and Years of Life Lost (YLLs) from DN linked to excessive intake of SSBs in regions categorized by Socio-Demographic Index (SDI), we employed the Age-Standardized Rate (ASR) and assessed the annual percentage change (EAPC) (45, 46). Standardizing data is essential for comparing different populations with distinct age structures or for examining the same population across different time periods, given that the age profile evolves (47, 48). The Age-Standardized Incidence Rate (ASIR) and Age-Standardized Death Rate (ASDR) per 100,000 individuals were calculated by aggregating the products of age-specific rates, where dk represents the distribution of the chosen reference population’s age groups, and Bk denotes the age-specific rate. A weighted average of these rates was derived based on the age distribution, with the relevant formulas detailed:


$$\mathrm{ASR}=\frac{\sum_{k=1}^{n}\alpha_{k\beta_{k}}}{\sum_{k=1\beta_{k}}^{n}}\times 100,000$$

The EAPC and their 95% confidence intervals (Cls) are a comprehensive measure of ASR trends over a specific period, with lower bounds above 0points to an upward trend, whereas upper bounds below 0 points to a downward trend. We use a log-linear regression to calculate (where y = In [ASR], and x = calendar year), as follows:


y=a+βx+ε

EAPC=100%x(eβ−1)

The analysis of deaths, DALYs, YLDs, and YLLs across high and low SDI regions includes the Age-Standardized Rate (ASR) for the year 2021, the percentage change in cases, and the Estimated Annual Percentage Change (EAPC) from 1990 to 2021. An upward trend in the ASR of deaths, DALYs, YLDs, and YLLs is indicated by positive estimates for both the EAPC and the 95% confidence intervals (CIs), and conversely, a downward trend is indicated by negative values. Furthermore, Pearson correlation analysis was conducted to estimate the correlation coefficients and *p*-values. All data visualization and statistical analyses were conducted using GraphPad Prism (version 8.02) and R software (version 3.5.2). The ARIMA model is a differential integrated moving average autoregressive model, also known as an integrated moving average autoregressive model, which is one of the time series forecasting analysis methods. In ARIMA (p, d, q), AR is “autoregressive” and p is the number of autoregressive terms. MA is the “moving average,” q is the number of terms in the moving average, and d is the number of differences (order) made to make it a stationary sequence. The model fitting was evaluated by coefficient of, Mean Absolute Error (MAE), and Root Mean Squared Error (RMSE). The lower The MAE and RMSE values, the better the model. The specific values and related images for ARIMA (p, d, q) can be found in the Supplementary Data Sheets 1–9.

## 3 Results

### 3.1 Diabetic nephropathy caused by excessive intake of SSBs in high SDI regions

**Figure 1:**
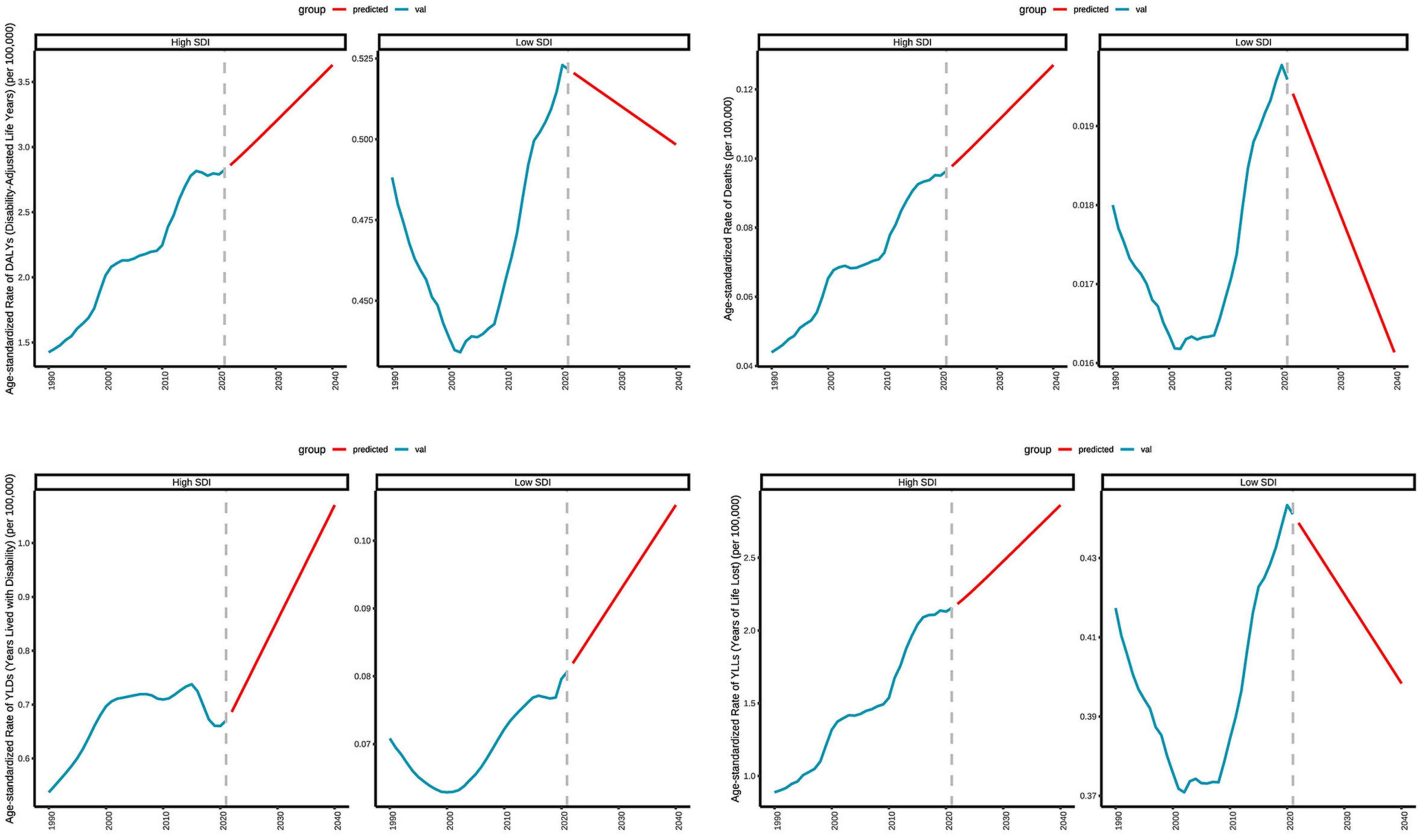
Distribution of deaths, DALYs, YLDs, and YLLs attributable to diabetic nephropathy caused by excessive sugary drink intake in high SDI regions in 2021 by age and sex.

As shown in Figure 1 and Table 1, this image displays the burden analysis results of type 2 DN caused by excessive intake of SSBs in high SDI regions in2021. The results indicate that the number of YLDs exhibit a certain trend of change across different age groups. Starting from the 25- to 29-year-old age group, YLDs values gradually increase with age, reaching to1201 in the 50- to 54-year-old age group. Subsequently, there is some fluctuation in the higher age groups, but overall, the values remain at a relatively high level. There are certain differences in YLDs values between males and females in each age group, with males having slightly higher YLDs values than females in most age groups. Notably, the peaks for the age groups 65 to 69 year old and 75 to 79 year old are 1,286 and 1,060 respectively, indicating that the loss of life span due to this disease is more severe in these age groups in 2021. Moreover, the number of YLLs for males are significantly higher than those for females in most age groups. The number of deaths shows a certain increasing trend across different age groups, especially in the age group of 65 years and above, where the increase in the number of deaths is more evident. Additionally, the number of male deaths is higher than that of females in each age group, demonstrating that this disease poses a greater threat to the lives of people in the older age group and that males face a higher risk of death. The number of DALYs show a similar trend of change to YLDs and YLLs across different age groups. The relative peaks in the 50- to 54-year-old and 65- to 69-year-old age groups are 3,518 and 4,366, respectively. The DALYs values for males are generally higher than those for females in each age group, reflecting that the overall health loss caused by this disease to the male population in high SDI regions is more severe.

**Table 1:** All-age cases and age-standardized deaths, DALYs, YLDs, and YLLs rates in 2021 for DN caused by excessive sugary drink intake in high SDI regions.

Measure	All-ages cases total	Male	Female	Age-standardized rates per 100,000 people total	Male	Female
Deaths (95% UI)	2,132 (1,117–3,211)	1,009 (525–1,503)	1,122 (567–1774)	0.096 (0.051–0.144)	0.106 (0.055–0.158)	0.088 (0.044–0.136)
DALYs (95% UI)	52,574 (27,968–77,829)	26,159 (13,500–39,254)	26,415 (13,928–39,780)	2.824 (1.462–4.174)	2.984 (1.53–4.449)	2.698 (1.418–4.078)
YLDs (95% UI)	11,564 (5,376–18,993)	5,536 (2,593–9,017)	6,028 (2,823–10,214)	0.67 (0.312–1.103)	0.66 (0.311–1.069)	0.686 (0.318–1.153)
YLLs (95% UI)	41,010 (21,522–60,935)	20,622 (10,686–30,881)	20,388 (10,260–31,271)	2.154 (1.123–3.189)	2.324 (1.197–3.484)	2.012 (1.029–3.039)

### 3.2 Diabetic nephropathy caused by excessive intake of SSBs in low SDI regions

**Figure 2:**
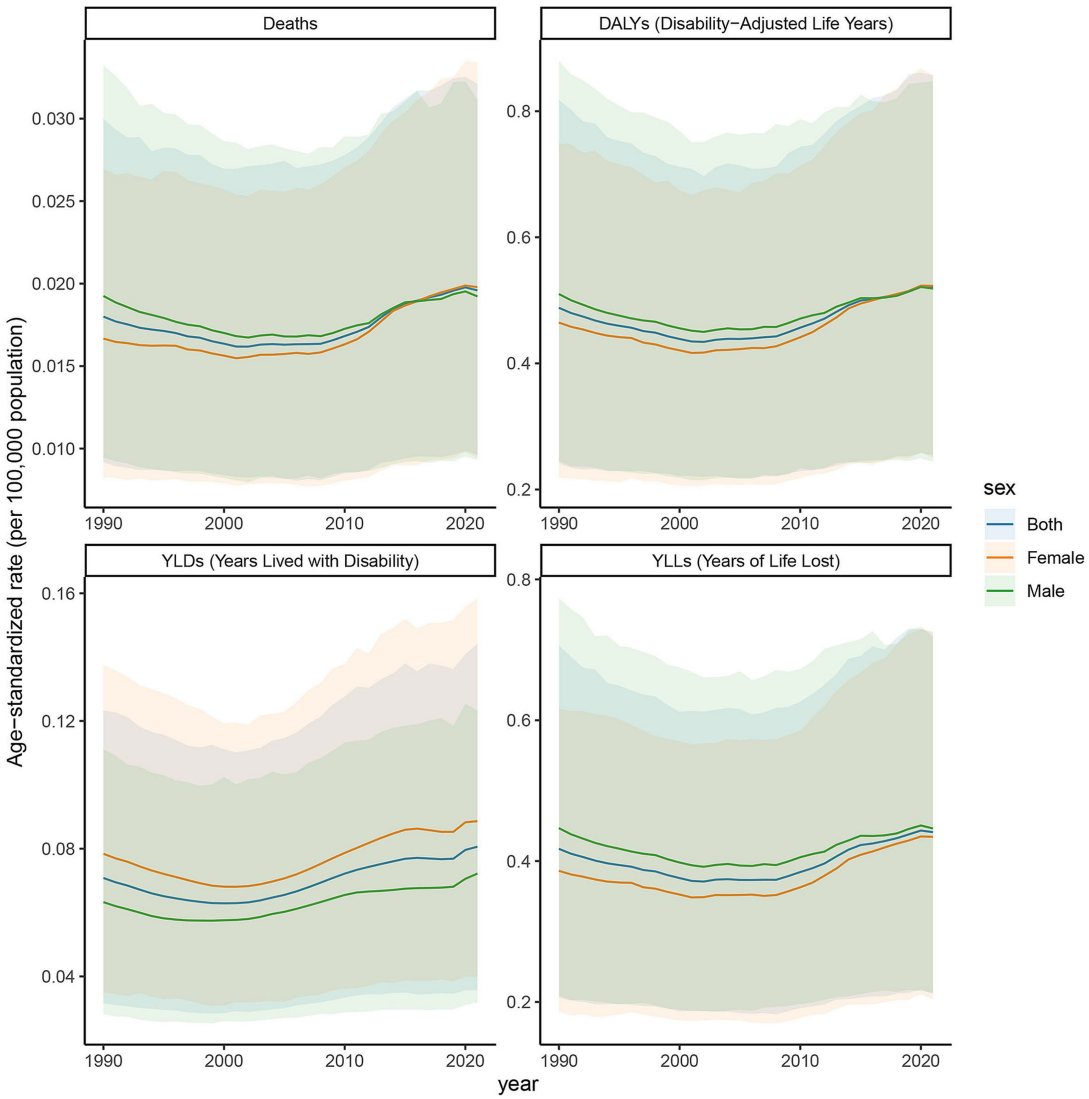
Distribution of deaths, DALYs, YLDs, and YLLs attributable to diabetic nephropathy caused by excessive sugary drink intake in low SDI regions in 2021 by age and sex.

As depicted in Figure 2 and Table 2, this image presents the burden analysis results of type 2 DN caused by excessive intake of SSBs in low SDI regions in 2021. The findings reveal the following that from the 25- to 29-year-old age group to the 95 year old and above age group, the number of YLDs show an overall upward trend. There is a more pronounced increase in the 55 to 59 year old and 70- to 74-year-old age groups. The differences in YLDs values between males and females across various age groups are relatively small, but males have slightly higher values than females in some age groups. Relative peaks occur in the 60 to 64 year old and 75- to 79-year-old age groups, which are 47 and 8. The YLLs values for males are higher than those for females in most age groups, indicating that the impact of this disease on life span loss in low SDI regions is more significant for males. The number of deaths shows an increasing trend across different age groups, with a more evident increase in the age group of 65 years and above. The number of male deaths is higher than that of females in each age group, suggesting that the threat of this disease to male life and health in low SDI regions is more prominent. The number of DALYs exhibit a similar trend to YLDs and YLLs across different age groups. Relative peaks appear in the 55 to 59 year old and 65- to 69-year-old age groups which are 178 and 158. The number of DALYs for males are generally higher than those for females in each age group, reflecting that the overall health loss caused by this disease to the male population in low SDI regions is more severe.

**Table 2:** All-age cases and age-standardized deaths, DALYs, YLDs, and YLLs rates in 2021 for DN caused by excessive sugary drink intake in low SDI regions.

Measure	All-ages cases total	Male	Female	Age-standardized rates per 100,000 people total	Male	Female
Deaths (95% UI)	87 (42–142)	43 (20–69)	44 (21–74)	0.02 (0.01–0.032)	0.019 (0.009–0.031)	0.02 (0.009–0.033)
DALYs (95% UI)	2,867 (1,372–4,659)	1,419 (673–2,337)	1,449 (692–2,312)	0.522 (0.254–0.857)	0.519 (0.244–0.847)	0.523 (0.25–0.857)
YLDs (95% UI)	504 (220–898)	223 (99–392)	281 (124–497)	0.081 (0.036–0.144)	0.072 (0.032–0.123)	0.089 (0.04–0.158)
YLLs (95% UI)	2,363 (1,122–3,942)	1,196 (555–1980)	1,167 (543–1939)	0.441 (0.211–0.725)	0.446 (0.213–0.718)	0.434 (0.204–0.726)

### 3.3 Age-specific rates of deaths, DALYs, YLDs, and YLLs in high SDI regions

**Figure 3:**
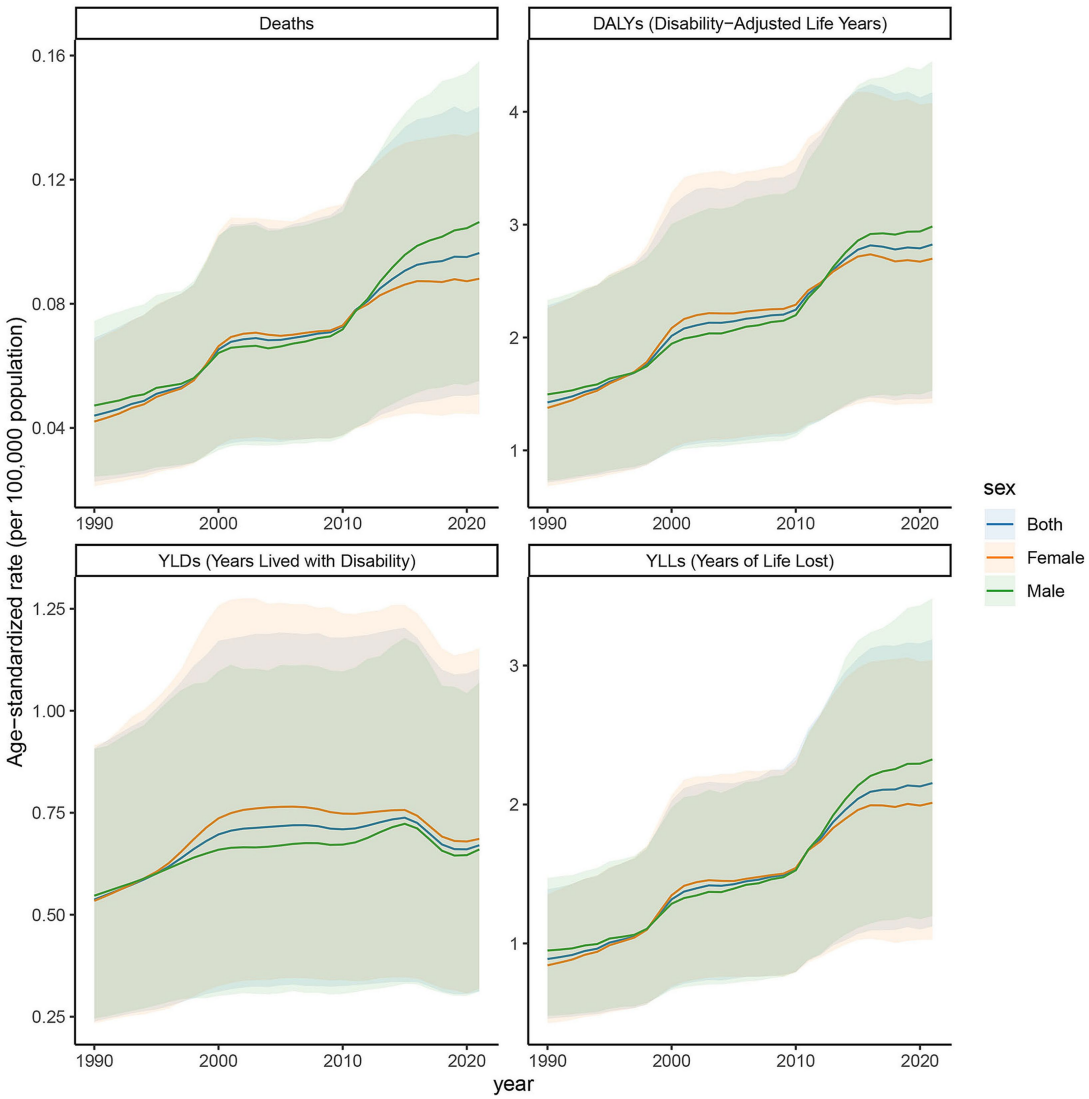
Age-specific rates of deaths, DALYs, YLDs, and YLLs attributable to diabetic nephropathy caused by excessive sugary drink intake in high SDI regions in 2021 by age and sex.

The age-specific rates illustrated in Figure 3 reveal pronounced sex and age-related differences across deaths, DALYs, YLDs, and YLLs for type 2 DN caused by excessive intake of SSBs in high SDI regions in 2021. The age-specific rate of YLDs in high SDI regions shows a certain trend of change across different age groups. Starting from the 25- to 29-year-old age group, YLDs values gradually increase with age, reaching to 69 in the 50- to 54-year-old age group. Subsequently, there is some fluctuation in the higher age groups, but overall, the values remain at a relatively high level. There are certain differences in YLDs values between males and females in each age group, with males having slightly higher YLDs values than females in most age groups. The age-specific rate of YLLs is widely distributed across various age groups, with relatively high values ranging from the 25- to 29-year-old age group to the 95 year old and above age group. Notably, there are relatively pronounced peaks in the 65 to 69 year old and 75- to 79-year-old age groups, which are 7.203 per 100,000 and 12.337 per 100,000, indicating that the loss of life span due to this disease is more severe in these age groups. Moreover, the YLLs values for males are significantly higher than those for females in most age groups. The age-specific death rate shows a certain increasing trend across different age groups, especially in the age group of 65 years and above, where the increase is more evident. The age-specific rate of DALYs shows a similar trend of change to YLDs and YLLs across different age groups. The relative peaks in the 50 to 54 year old and 65- to 69-year-old age groups which are 6 per 100,000 and 9 per 100,000. The DALYs values for males are generally higher than those for females in each age group, reflecting that the overall health loss caused by this disease to the male population in high SDI regions is more severe.

### 3.4 Age-specific rates of deaths, DALYs, YLDs, and YLLs in low SDI regions

**Figure 4:**
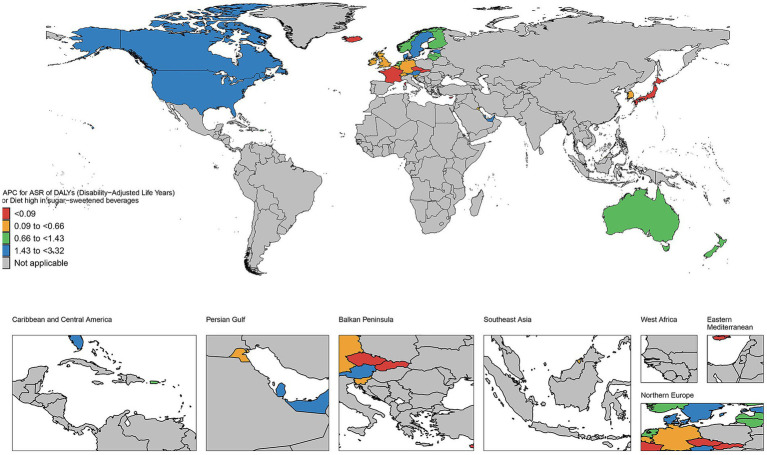
Age-specific rates of deaths, DALYs, YLDs, and YLLs attributable to diabetic nephropathy caused by excessive sugary drink intake in low SDI regions in 2021 by age and sex.

The age-specific rates illustrated in Figure 4 reveal pronounced sex and age-related differences across deaths, DALYs, YLDs, and YLLs for type 2 DN caused by excessive intake of SSBs in low SDI regions in 2021. The age-specific rate of YLDs in low SDI regions shows an upward trend from the 25- to 29-year-old age group to the 95 year old and above age group. There is a more noticeable increase in the 55 to 59 year old and 70- to 74-year-old age groups, which are, respectively, 0.234 per 100,000 and 0.263 per 100,000. Relative peaks occur in the 60 to 64 year old and 75- to 79-year-old age groups, which are 0.24 per 100,000 and 0.29 per 100,000. The YLLs values for males are higher than those for females in most age groups, indicating that the impact of this disease on life span loss in low SDI regions is more significant for males. The age-specific rate of death shows an increasing trend across different age groups, with a more evident increase in the age group of 65 years and above. The number of male deaths is higher than that of females in each age group, suggesting that the threat of this disease to male life and health in low SDI regions is more prominent. The age-specific rate of DALYs exhibits a similar trend to YLDs and YLLs across different age groups. Relative peaks appear in the 55 to 59 year old and 65- to 69- year-old age groups, which are 1.651 per 100,000 and 2.143 per 100,000. The DALYs values for males are generally higher than those for females in each age group, reflecting that the overall health loss caused by this disease to the male population in low SDI regions is more severe.

### 3.5 Trends in age-standardized rates of deaths, DALYs, YLDs, and YLLs in high SDI regions, 1990–2021

**Figure 5:**
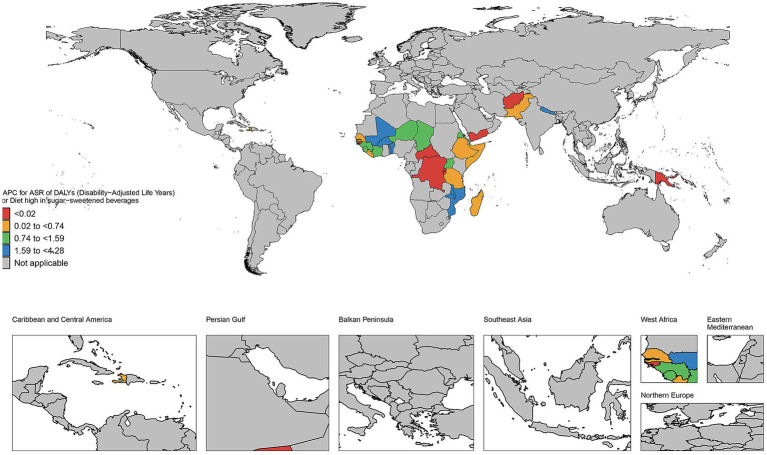
Trends in age-standardized rates of deaths, DALYs, YLDs, and YLLs attributable to diabetic nephropathy caused by excessive sugary drink intake in high SDI regions, 1990–2021.

The age-standardized rates illustrated in Figure 5 reveal pronounced sex- and age-related differences in deaths, DALYs, YLDs, and YLLs due to DN resulting from excessive intake of SSBs in high SDI regions from 1990 to 2021. In these regions, the ASR of the burden of chronic kidney disease attributed to type 2 diabetes mellitus, caused by excessive intake of SSBs, exhibited an increasing trend from 2019 to 2021. Specifically, the ASR for deaths rose from 0.044 (95% CI: 0.023–0.069) in 2019 to 0.096 (95% CI: 0.051–0.144) in 2021. The ASR for DALYs increased from 1.425 (95% CI: 0.715–2.284) to 2.824 (95% CI: 1.462–4.174). For YLDs, the ASR increased from 0.537 (95% CI: 0.239–0.908) to 0.670 (95% CI: 0.312–1.103). Additionally, the ASR for YLLs climbed from 0.888 (95% CI: 0.458–1.390) to 2.154 (95% CI: 1.123–3.189). Among females, the ASR for deaths was 0.042 (95% CI: 0.021–0.068) in 2019 and increased to 0.088 (95% CI: 0.044–0.136) in 2021. For males, the ASR was 0.047 (95% CI: 0.024–0.074) in 2019 and rose to 0.106 (95% CI: 0.055–0.158) in 2021. This indicates a significant increase in the disease burden in high SDI regions over these 2 years. From 1990 to 2021, the age-standardized rate of YLDs in high SDI regions exhibited an upward trend. The YLDs for both males and females have increased, with a more pronounced rise observed in females compared to males. Similarly, the age-standardized rate of YLLs exhibited an upward trend from 1990 to 2021. The YLLs for both sexes have risen, with females experiencing a higher increase than males. The age-standardized rate of deaths has gradually ascended from 1990 to 2021. In 1990, the deaths was notably low; however, after more than 30 years of development, it had increased substantially by 2021. The deaths for both males and females have risen, with the increase in female mortality rates occurring at a relatively faster pace. Moreover, the age-standardized rate of DALYs has continued to rise from 1990 to 2021. The DALYs was low in 1990, but by 2021, it had significantly increased, indicating a continuous rise in overall health loss due to this disease in SDI regions. The DALYs for both males and females have increased, with a more significant rise in females, particularly among middle-aged and older adult populations.

### 3.6 Trends in age-standardized rates of deaths, DALYs, YLDs, and YLLs in low SDI regions, 1990–2021

**Figure 6:**
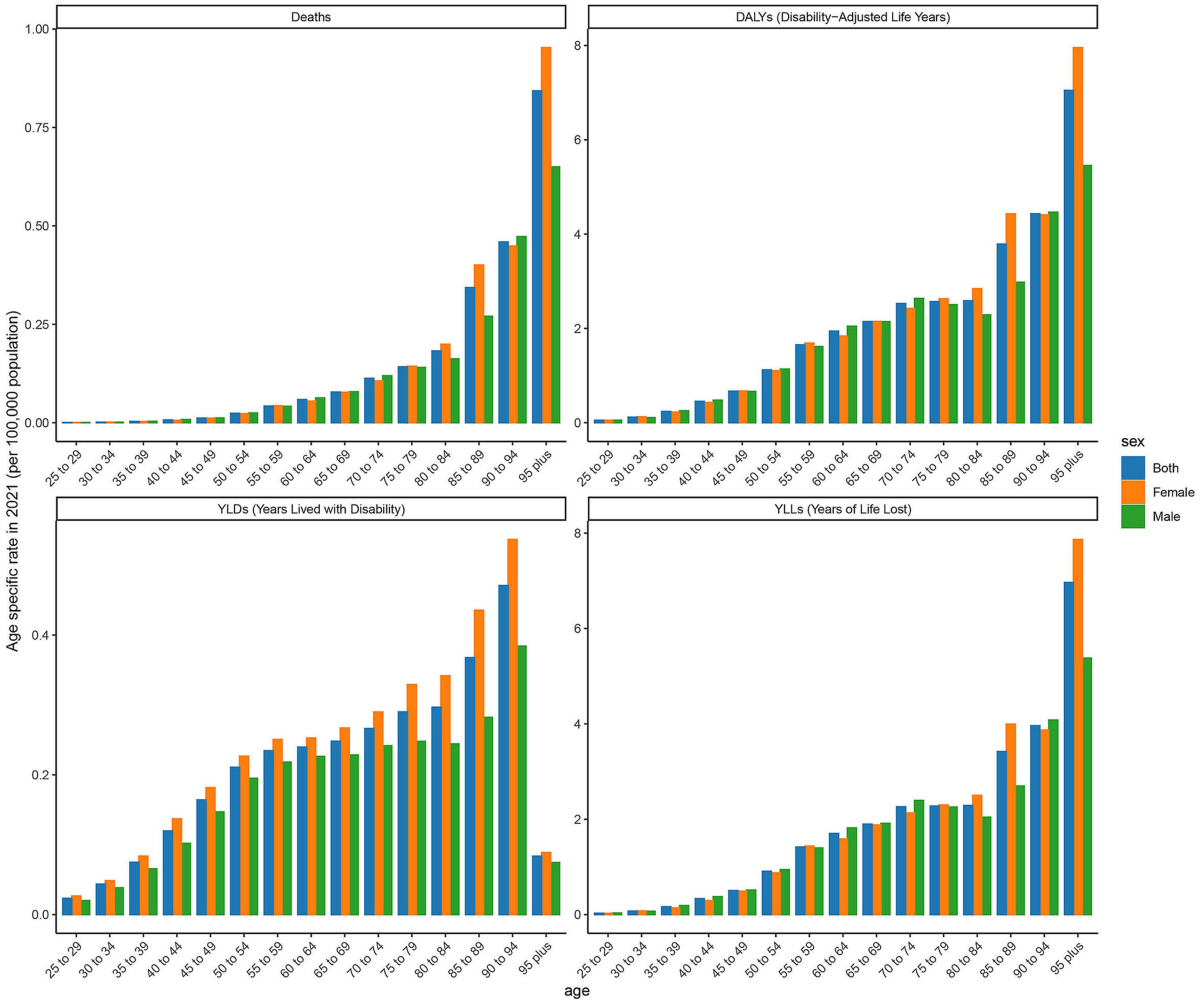
Trends in age-standardized rates of deaths, DALYs, YLDs, and YLLs attributable to diabetic nephropathy caused by excessive sugary drink intake in low SDI regions, 1990–2021.

The age-standardized rates illustrated in Figure 6 reveal pronounced sex and age-related differences in deaths, DALYs, YLDs, and YLLs due to DN resulting from excessive intake of SSBs in low Socio-Demographic Index (SDI) regions from 1990 to 2021. Overall, from 2019 to 2021, the age-standardized rates (ASR) for deaths, DALYs, YLDs, and YLLs attributed to this cause in low SDI regions demonstrated an upward trend. Specifically, the ASR for deaths increased from 0.018 (95% CI: 0.009–0.03) in 2019 to 0.02 (95% CI: 0.01–0.032) in 2021; the ASR for DALYs rose from 0.488 (95% CI: 0.242–0.818) to 0.522 (95% CI: 0.254–0.857); the ASR for YLDs increased from 0.071 (95% CI: 0.032–0.123) to 0.081 (95% CI: 0.036–0.144); and the ASR for YLLs grew from 0.417 (95% CI: 0.206–0.706) to 0.441 (95% CI: 0.211–0.725). The increase was relatively more pronounced among females, with the ASR for deaths rising from 0.017 (95% CI: 0.008–0.027) to 0.02 (95% CI: 0.009–0.033), whereas the change among males was comparatively smaller. From 1990 to 2021, the YLDs for both males and females increased, with a more substantial rise observed in females, particularly in younger age groups. Similarly, YLLs for both sexes also increased, with females exhibiting a higher increase than males, particularly in younger cohorts. In 1990, the deaths was low; however, by 2021, it had risen significantly, indicating a growing threat posed by this disease to individuals in low SDI regions. The age-standardized DALY rate continued to rise from 1990 to 2021, reflecting a significant increase in overall health loss attributed to this disease in low SDI regions. Both males and females experienced an increase in DALY rates, with the rise being more pronounced in females than in males.

### 3.7 The EAPC in age-standardized rates of DALYs in countries and territories of high and low SDI regions from 1990 to 2021

**Figure 7:**
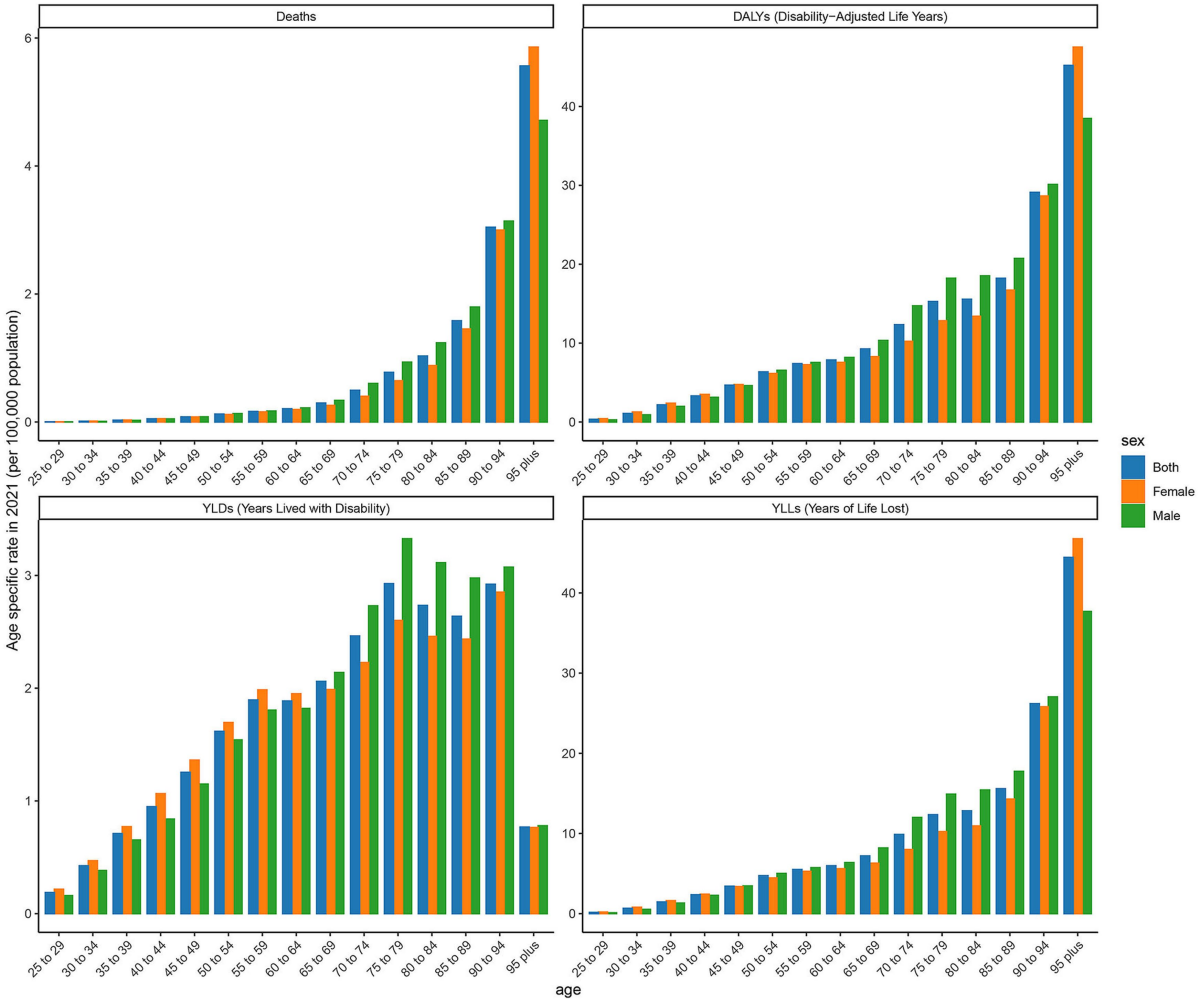
The EAPC in age-standardized rates of DALYs in countries and territories of high SDI regions from 1990 to 2021.

**Figure 8:**
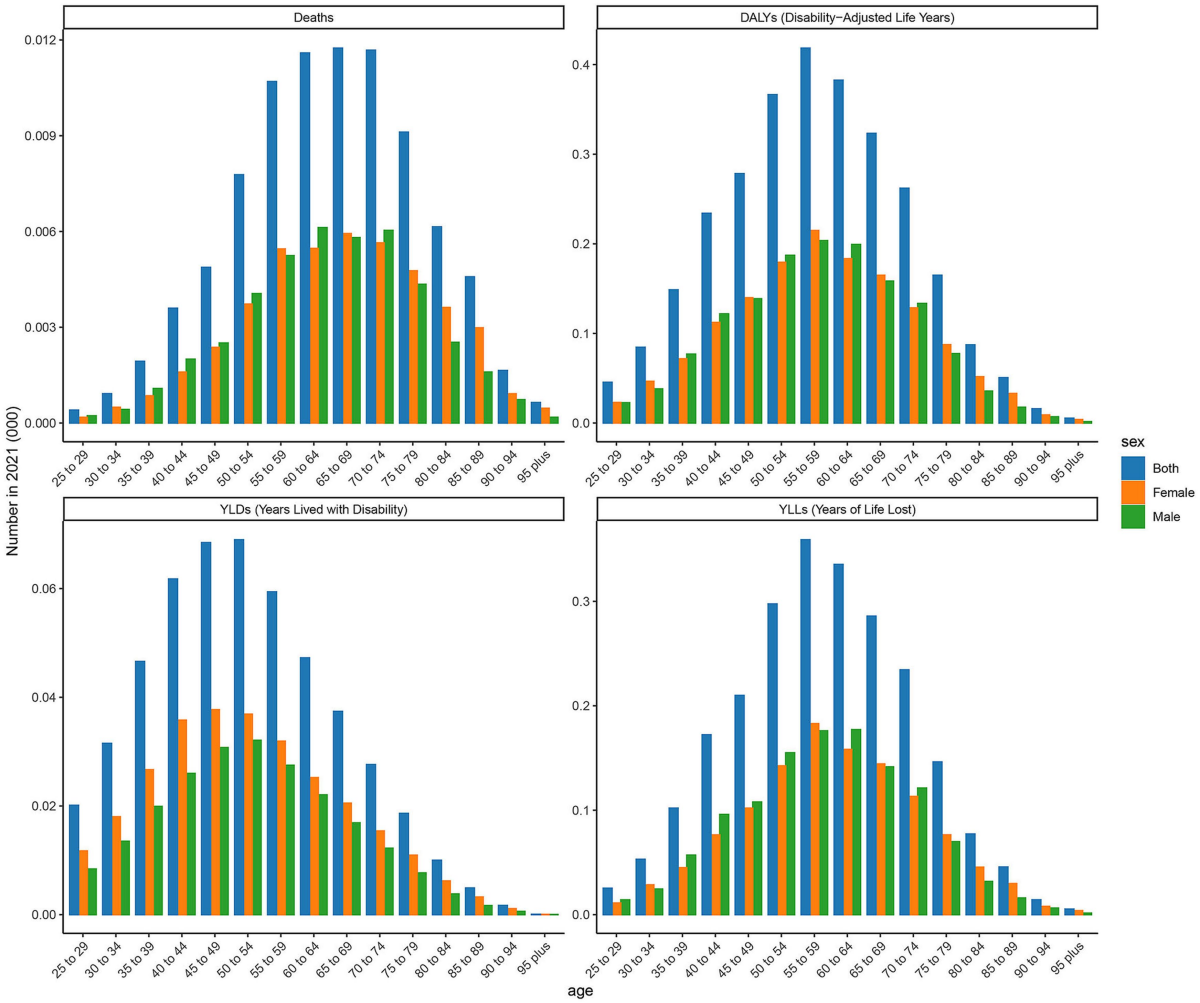
The EAPC in age-standardized rates of DALYs in countries and territories of low SDI regions from 1990 to 2021.

As illustrated in Figure 7, the number of deaths has increased in most countries and regions during this period. For instance, in the United States, the number of deaths rose from 223 in 1990 to 1,300 in 2021, representing nearly a five-fold increase. Similar upward trends are observed in other countries such as Canada, Australia, and the United Kingdom. The DALYs in high SDI regions have generally increased. Taking the United States as an example, its DALYs increased from 7,508 in 1990 to 33,065 in 2021, an increase of approximately 340%. Significant growth trends are also evident in other countries like Canada, Australia, and the United Kingdom. The YLDs have similarly increased across most countries and regions. For example, in the United States, YLDs grew from 2,573 in 1990 to 5,785 in 2021, an increase of about 125%. Comparable growth trends are observed in other countries such as Canada, Australia, and the United Kingdom. Additionally, YLLs have significantly increased in most countries and regions. For instance, YLLs in the United States surged from 4,935 in 1990 to 27,281 in 2021, representing an increase of approximately 453%. Other countries, including Canada, Australia, and the United Kingdom, also exhibit significant upward trends. As depicted in Figure 8, the number of deaths, DALYs, YLDs, and YLLs have all increased in most countries. The deaths has risen in many nations during this period, particularly in several African countries, including Burkina Faso, Chad, and the Central African Republic. In terms of DALYs, values have increased in nearly all countries, reflecting the growing overall impact of the disease on population health. Notably, the rise in DALYs in Niger and Burundi indicates significant challenges these countries face in addressing the disease. The trends observed in YLDs and YLLs are similar; the increase in YLDs signifies a growing number of individuals living with disabilities due to illness, while the rise in YLLs suggests a worsening situation regarding premature deaths caused by illness. Particularly alarming is the substantial increase in YLLs in countries such as Guinea-Bissau and Madagascar, highlighting the severe impact of the disease on life expectancy.

### 3.8 Predicted trends of age-standardized rates of deaths, DALYs, YLDs, and YLLs in high and low SDI regions over 20 years (2021–2041)

**Figure 9:**
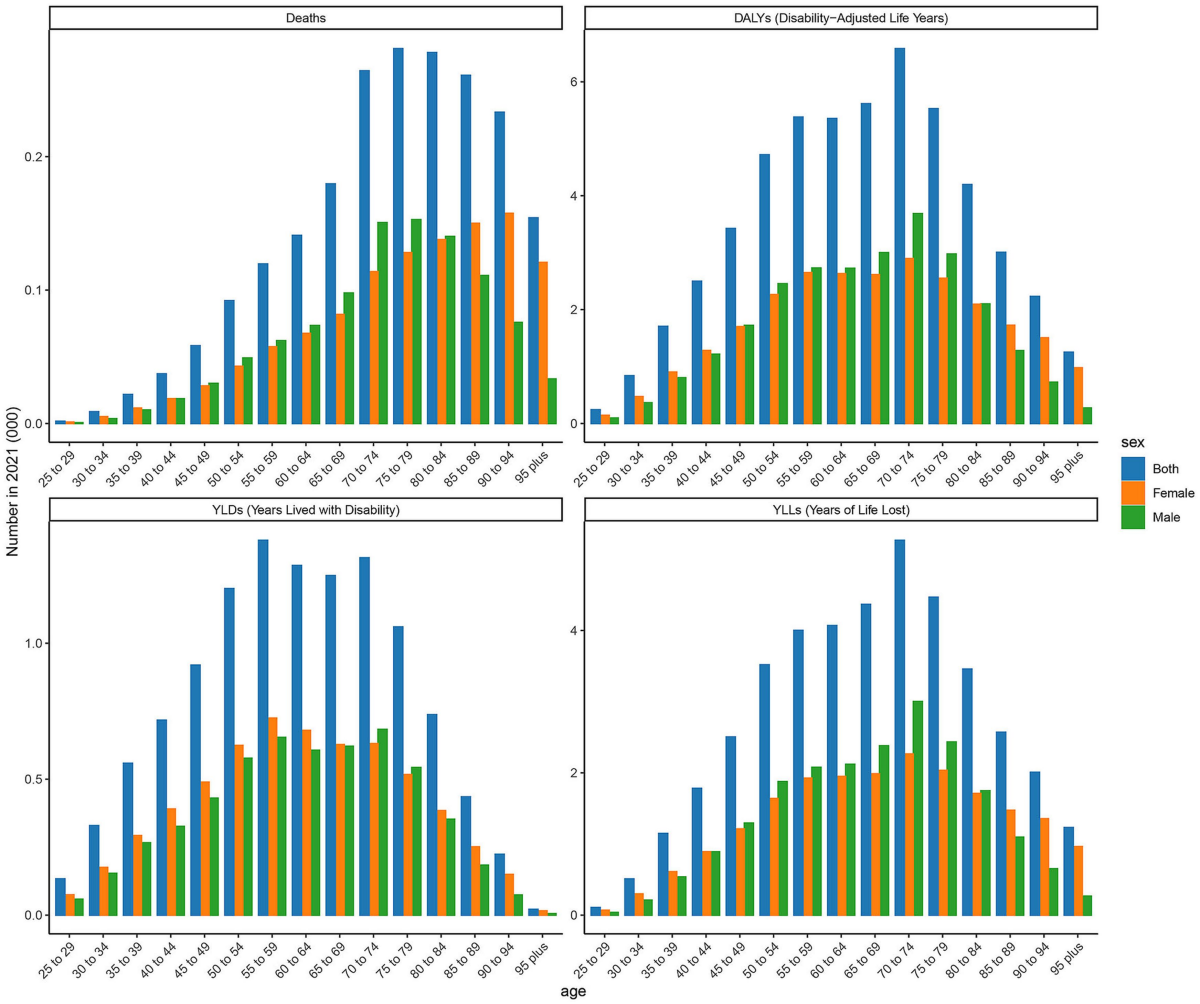
Predicted trends of age-standardized rates of deaths, DALYs, YLDs, and YLLs in high and low SDI regions over 20 years (2021–2041).

Figure 9 presents the actual data and projected trends of age-standardized rates in high and low SDI regions from 1990 to 2020, along with projections for 2021 to 2040. In high SDI regions, the mortality rate exhibits a consistent upward trend, increasing from 0.0978 per 100,000 in 2022 to 0.1270 per 100,000 in 2040. This trend indicates that the number of deaths attributable to DN resulting excessive intake of SSBs will continue to rise in high SDI regions in the future. DALYs also demonstrate a year-on-year increase, rising from 2.8610 per 100,000 in 2022 to 3.6290 per 100,000 in 2040, suggesting a growing health burden associated with this disease in these regions. In high SDI regions with advanced healthcare, longer life expectancy increases the proportion of older adult individuals. This aging population experiences physical decline, raising the risk of chronic diseases like cardiovascular illnesses, diabetes, and cancers, which are key contributors to and DALY increases. YLDs increased from 0.6865 per 100,000 in 2022 to 1.0704 per 100,000 in 2040, reflecting an annual increase in YLLs. The YLLs rose from 2.1831 per 100,000 in 2022 to 2.8615 per 100,000 in 2040, indicating a rise in YLLs due to premature death. Conversely, in low SDI regions, the deaths shows a consistent downward trend, decreasing from 0.0194 per 100,000 in 2022 to 0.0161 per 100,000 in 2040. This suggests a decline in the number of deaths due to type 2 diabetic nephropathy linked to excessive intake of SSBs in low SDI regions in the future. DALYs in these regions also exhibit a decreasing trend, declining from 0.5205 per 100,000 in 2022 to 0.4984 per 100,000 in 2040, indicating a gradual reduction in the health burden from the disease. YLDs increased from 0.0819 per 100,000 in 2022 to 0.1052 per 100,000 in 2040, demonstrating an annual increase in YLLs due to disability. In contrast, YLLs decreased from 0.4389 per 100,000 in 2022 to 0.3984 per 100,000 in 2040, reflecting a reduction in YLLs due to premature death.

### 3.9 Predicted trends of age-standardized rates of DALYs in low SDI and high SDI countries over 20 years (2021–2041)

**Figure 10:**
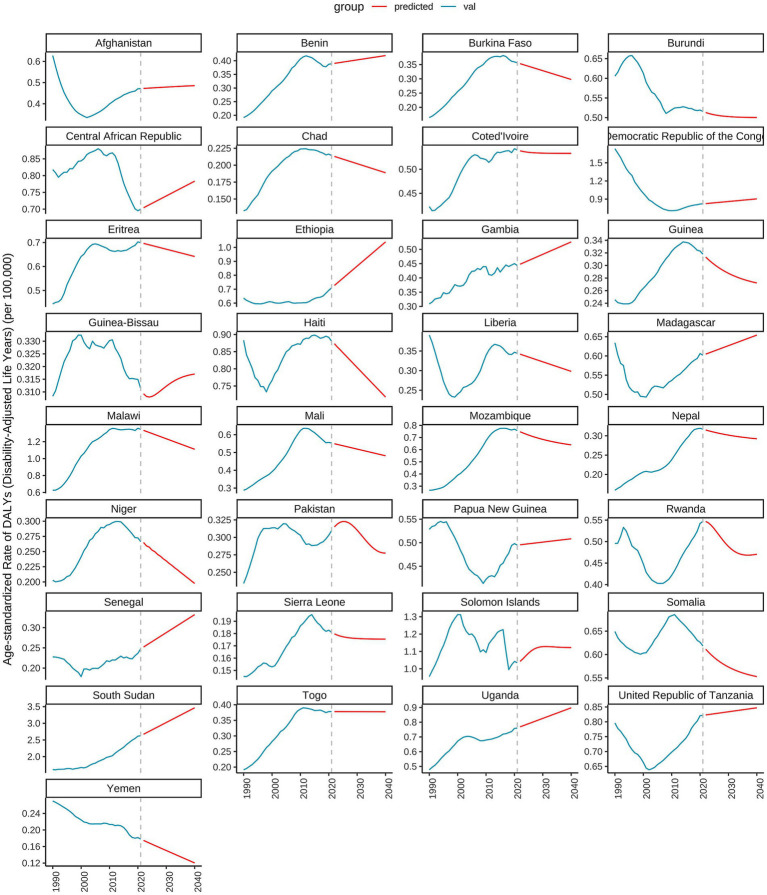
Predicted trends of age-standardized rates of DALYs in high SDI countries over 20 years (2021–2041).

**Figure 11:**
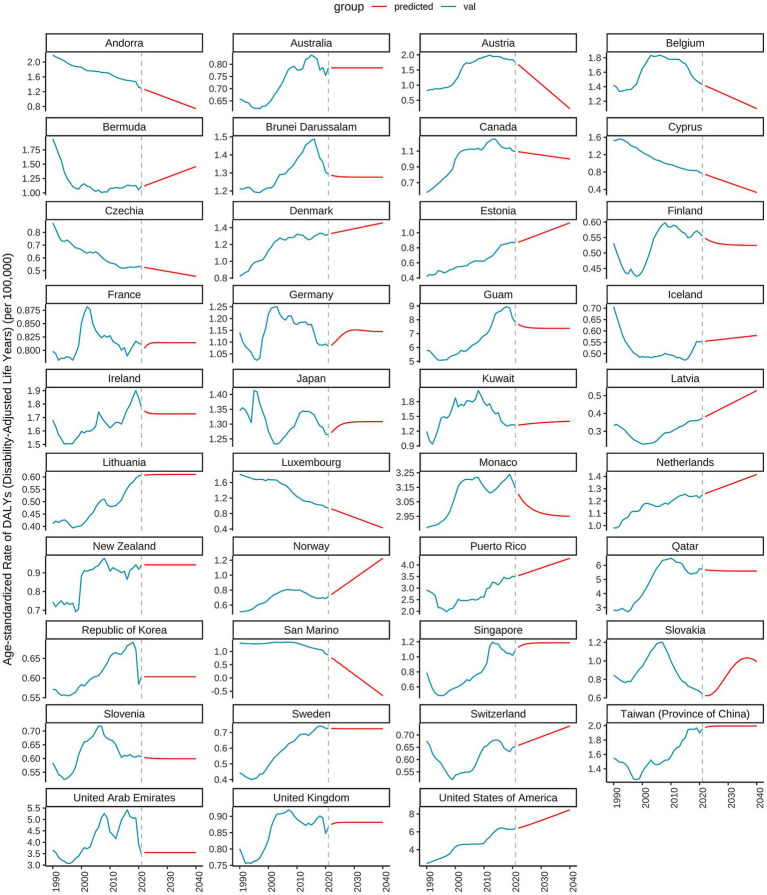
Predicted trends of age-standardized rates of DALYs in low SDI countries over 20 years (2021–2041).

As illustrated in Figures 10, 11, the burden of DN attributable to excessive intake of SSBs in high SDI regions has exhibited divergent trends from 1990 to 2021. The age-standardized rate of DALYs reveals that certain countries, such as Slovakia, Australia, and Canada, have experienced an overall upward trend during this period, indicating an increased disease burden. In contrast, countries like Iceland and Japan have demonstrated relatively stable trends, with minimal changes in disease burden. Specifically, Slovakia reported a DALYs rate of 0.6247 in 2021, projected to rise to 0.9993 by 2040, indicating a significant upward trajectory. Conversely, Australia’s DALYs was 0.7853 in 2021, projected to remain unchanged by 2040, reflecting a relatively stable disease burden. Additionally, Canada recorded a DALYs of 1.0912 in 2021, with expectations of a decrease to 0.9993 by 2040, suggesting a downward trend. As shown in Figure 11, significant disparities exist in DALYs among various low SDI countries. For instance, Somalia’s DALYs was 0.6113 in 2022, anticipated to decrease slightly each year, reaching 0.5528 by 2040. A similar trend is observed in Niger, where the DALYs was 0.2649 in 2022 and is projected to decline to 0.1975 by 2040. This suggests a potential alleviation of the burden of DN due to excessive intake of SSBs in these countries over the coming decades. However, the outlook for some nations remains less optimistic. For example, Chad’s DALYs was 0.2129 in 2022, expected to decrease only slightly to 0.1888 by 2040, indicating a relatively heavy disease burden. In contrast, Ethiopia’s DALYs is projected to rise from 0.7271 in 2022 to 1.0392 in 2040, signaling greater health challenges for the country in the future.

## 4 Discussion

The studies have shown that in 2020, about 2.2 million new cases of T2DM globally were attributable to excessive intake of SSBs, accounting for 9.8% of all new T2DM cases (29). The link between SSBs and T2DM is well-established, as the high sugar content in these beverages can lead to weight gain, insulin resistance, and ultimately the development of T2DM (30–32). DN, a common and severe microvascular complication of T2DM, is a major cause of end-stage renal disease (ESRD) (33, 34). The development of DN is influenced by a combination of factors, including hyperglycemia, hypertension, dyslipidemia, and genetic predisposition (35, 36). In addition to SSB intake, overall dietary patterns may influence the burden of DN. For example, unhealthy dietary habits such as high-salt, high-fat (especially trans and saturated fats), and low-fiber diets may act in concert with SSB consumption to boost DN risk. Also, low physical activity can cut energy expenditure, raising the risk of weight gain and obesity, which in turn affects glycemic control and insulin sensitivity. Moreover, physical activity is linked to inflammatory and oxidative stress processes, which are also key in DN development. However, the role of SSB consumption in the development and progression of DN has gained increasing attention in recent years (37). Some studies have suggested that the high fructose content in SSBs may have a direct impact on renal function, leading to renal injury and the development of DN (38, 39).

This study comprehensively analyzed the burden of DN caused by SSBs in high and low SDI regions from 1990 to 2021 and projected the trends until 2040 using ARIMA models. The results revealed distinct patterns and trends in age-standardized rates of deaths, DALYs, YLDs, and YLLs between the two regions. In high SDI regions, the age-standardized rates of deaths, DALYs, YLDs, and YLLs showed a consistent upward trend from 1990 to 2021. The deaths increased significantly, indicating a growing number of deaths attributable to DN caused by SSBs. Similarly, the DALYs, which represent the overall health burden, also rose considerably, suggesting an increasing impact of the disease on the population’s health. The YLDs and YLLs increased as well, highlighting the rising burden of disability and premature death associated with this condition. In high SDI regions, the significant upward trend in the burden of DN caused by excessive intake of SSBs can be attributed to a combination of socioeconomic and healthcare factors, for example, people in high-SDI areas have a high standard of living, but health education about sugar-sweetened beverages is not widely available. Economically, as these regions develop and urbanize, there is a marked shift in lifestyle, including dietary changes and reduced physical activity. These changes drive up excessive intake of SSBs, boosting the incidence of diabetes and its complications. While healthcare resources are relatively abundant in high SDI regions, public awareness of SSBs’ harms remains low. Current public health policies also fall short in curbing SSB consumption. In low SDI regions, despite a growing DN burden, the pace of increase is slower and some indicators even show a decline. This might stem from enhanced public health policies in recent years. Some countries have intensified health education and publicity to boost awareness of healthy eating. Alongside, efforts to bolster healthcare service accessibility have improved early diabetes detection and diagnosis, partially mitigating DN’s growth. The projections for 2021 to 2040 indicated that these upward trends would continue, with further increases in deaths, DALYs, YLDs, and YLLs, suggesting a worsening public health challenge in the future. In contrast, low SDI regions exhibited different trends. Although the age-standardized rates of deaths, DALYs, YLDs, and YLLs also increased from 1990 to 2021, the pace of increase was relatively slower compared to high SDI regions. However, projections showed that from 2021 to 2040, the mortality rate and DALYs would start to decline, while YLDs would continue to rise slightly and YLLs would decrease. This suggests that while the burden of DN caused by SSBs remains a concern in low SDI regions, there may be potential for improvement in certain aspects such as reducing mortality and overall health burden through targeted interventions.

Our findings are consistent with previous research that has documented the increasing prevalence and burden of type 2 diabetes and its complications in high SDI regions (40). Studies have shown that excessive intake of SSBs is closely associated with the development of type 2 diabetes and its subsequent complications, including DN (41, 42). The rising trends in mortality, DALYs, YLDs, and YLLs observed in this study further emphasize the need for effective public health measures to address this issue. In low SDI regions, limited data has been available on the burden of DN caused by SSBs. Our study provides valuable insights into the trends and projections in these regions, which can inform future research and policy-making. The results of this study have important implications for public health policy. In high SDI regions, the continuous increase in the burden of DN caused by excessive sugary drink intake calls for urgent action. Policies aimed at reducing sugary drink consumption, such as taxation, marketing restrictions, and public education campaigns, should be strengthened (43). Additionally, improving access to healthcare services for early diagnosis and management of type 2 diabetes and its complications can help mitigate the rising burden of DN. In low SDI regions, while the overall burden may be lower compared to high SDI regions, the increasing trends still warrant attention. Public health interventions should focus on raising awareness about the risks of excessive intake of SSBs and promoting healthy dietary habits. Strengthening healthcare infrastructure and capacity building can also contribute to better prevention and management of DN in these regions (44).

In conclusion, this study provides a comprehensive analysis of the burden of type 2 diabetes nephropathy caused by excessive intake of SSBs in high and low SDI regions. The findings highlight the significant and increasing public health challenge posed by this condition, particularly in high SDI regions. In high SDI regions, higher taxes can be imposed on SSBs, with revenues used for public health education and diabetes prevention/treatment projects. For example, Mexico’s tax policy reduced SSB consumption. Limiting SSB advertising, especially to children/adolescents, in media and public places. Norway strictly restricts such advertising. A multidisciplinary DN management team should be established, including endocrinologists, nephrologists, dietitians, and nurses, to provide personalized treatment plans. In low SDI regions, while the trends are relatively more favorable, continued attention and appropriate measures are still required to prevent and control the burden of DN caused by excessive sugary drink intake, governments can encourage businesses to produce low- or no-sugar beverage alternatives via policies and tax incentives, increasing their market availability. Invest in primary healthcare by upgrading facilities, equipping them better, and training healthcare providers to enhance their DN management capabilities. Schools and families should work together to develop students’ healthy eating habits and reduce their consumption of SSBs.

## Data Availability

The original contributions presented in the study are included in the article/Supplementary material, further inquiries can be directed to the corresponding authors.

## Footnotes

^1^ http://ghdx.healthdata.org/gbd-results-tool
