# Supplementary figures and images for: Burden analysis of diabetic nephropathy caused by excessive intake of sugar-sweetened beverages in high and low SDI regions
Source: Front Public Health. 2025 Jun 27;13:1598278. doi: 10.3389/fpubh.2025.1598278 (PMC12248067; doi:10.3389/fpubh.2025.1598278)

Residuals from ARIMA(1,1,0) with drift

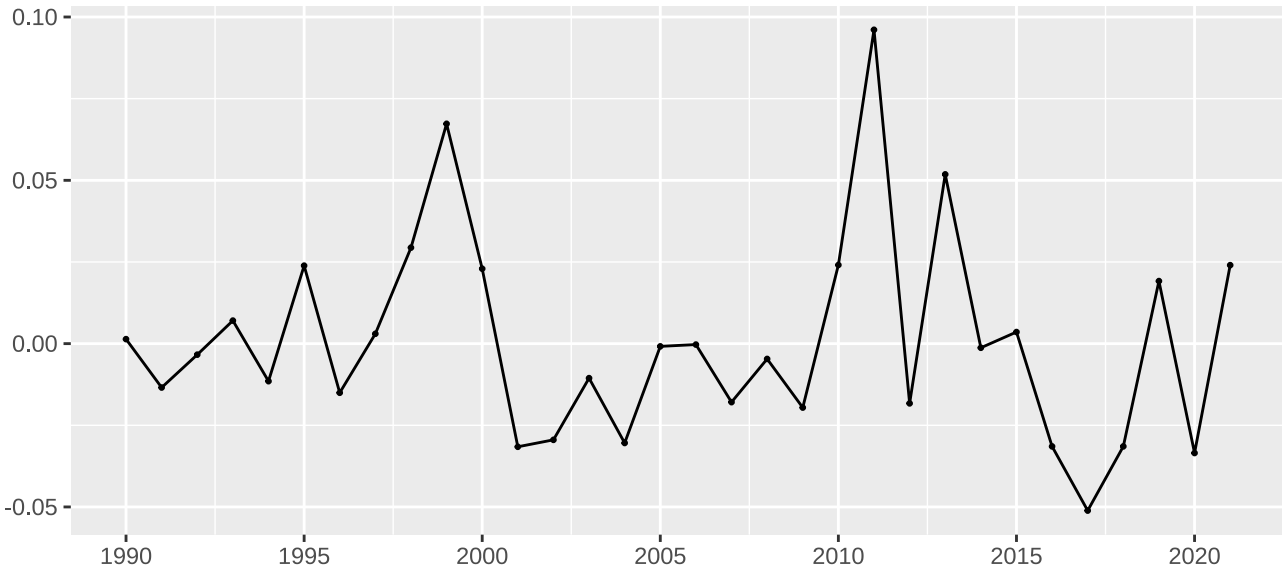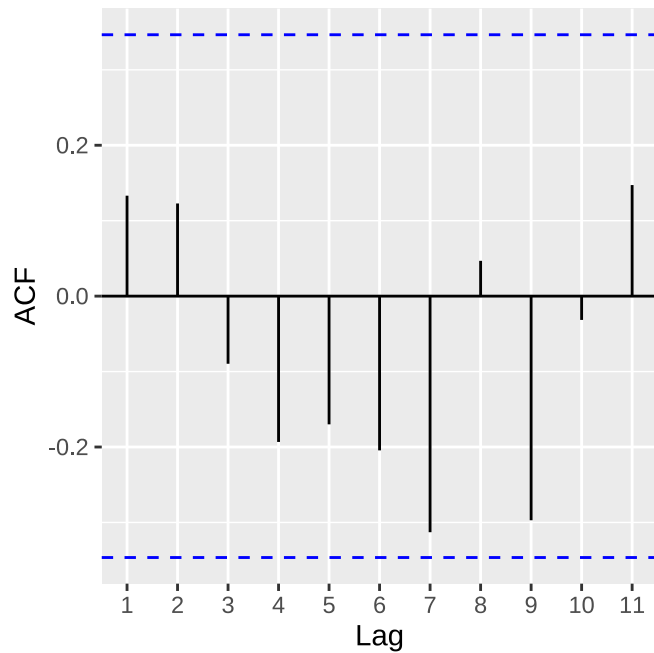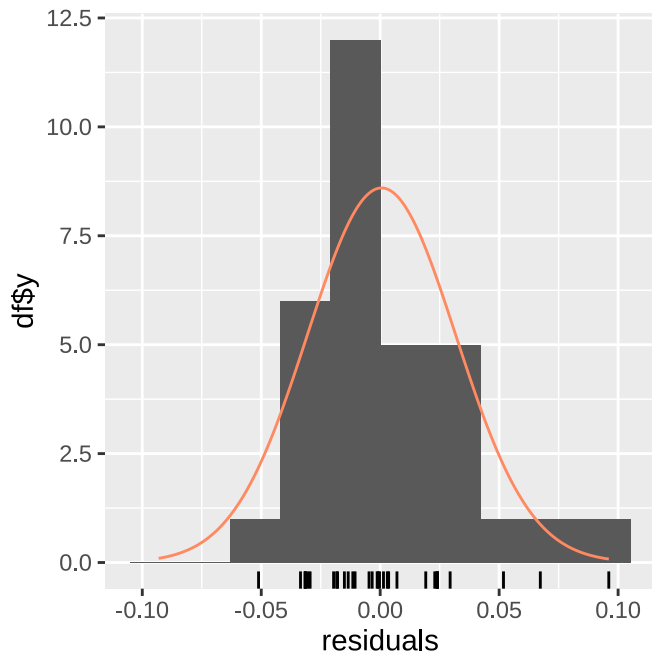

Supplement: Supplementary file 1 [file Data_Sheet_1.pdf]

Residuals from ARIMA(1,1,0) with drift

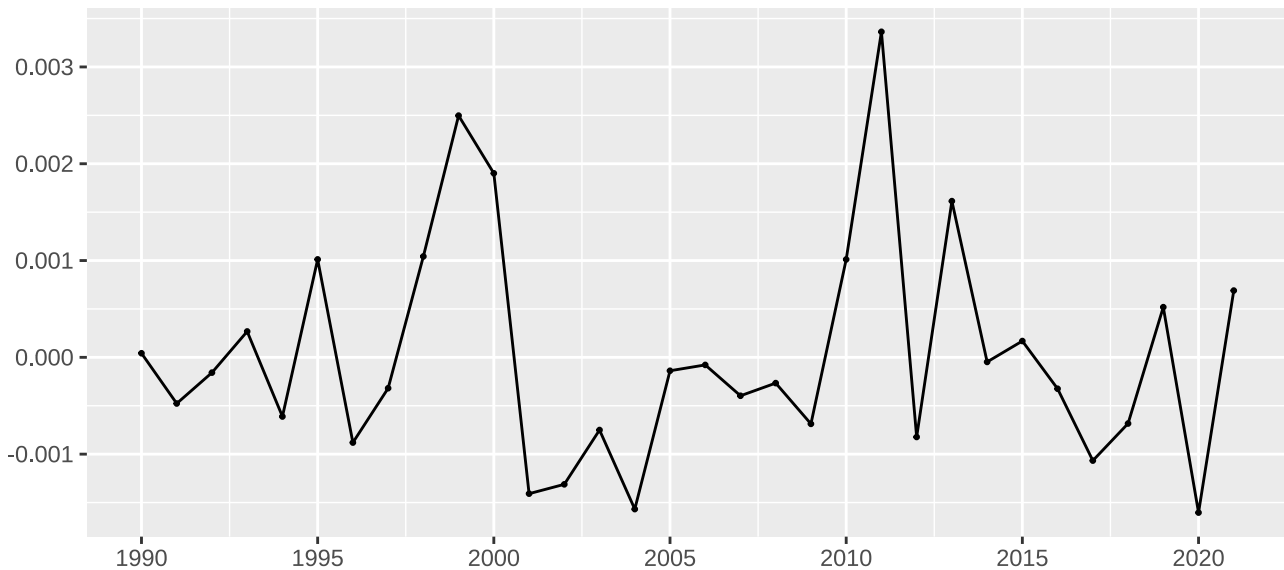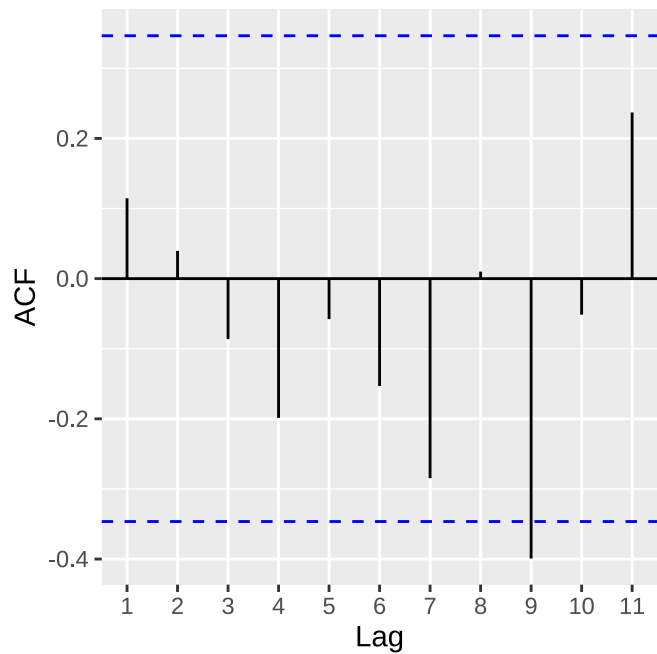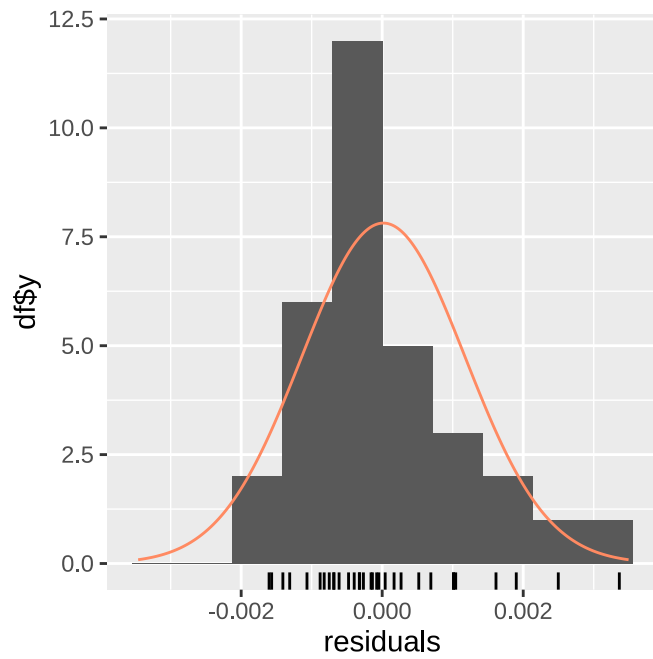

Supplement: Supplementary file 2 [file Data_Sheet_2.pdf]

Residuals from ARIMA(0,2,2)

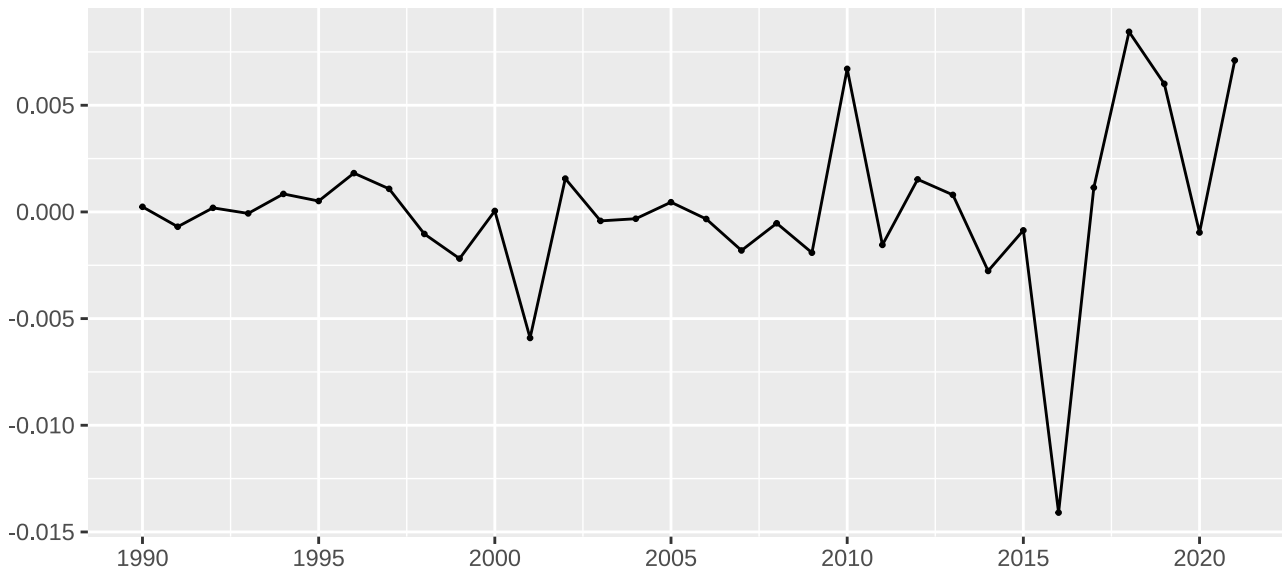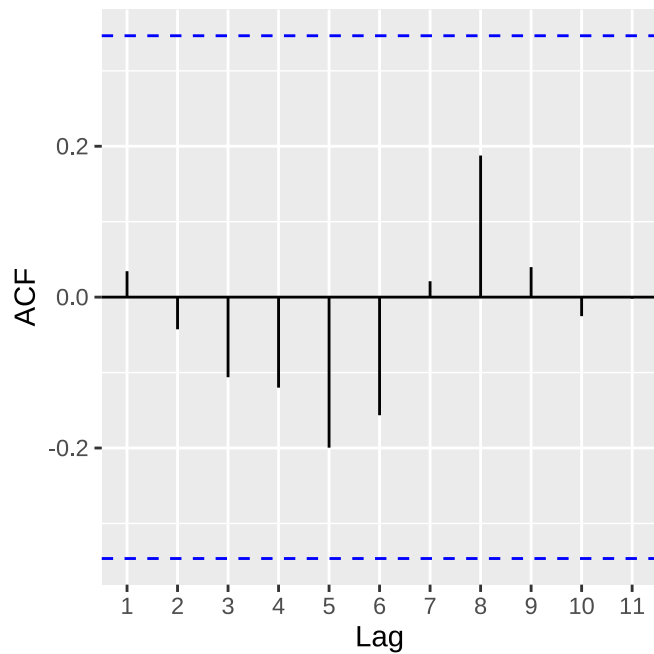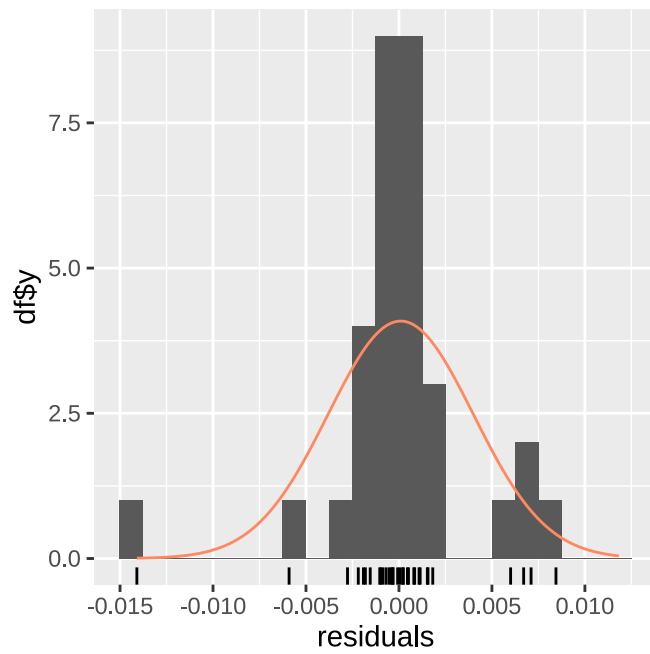

Supplement: Supplementary file 3 [file Data_Sheet_3.pdf]

Residuals from ARIMA(1,1,0) with drift

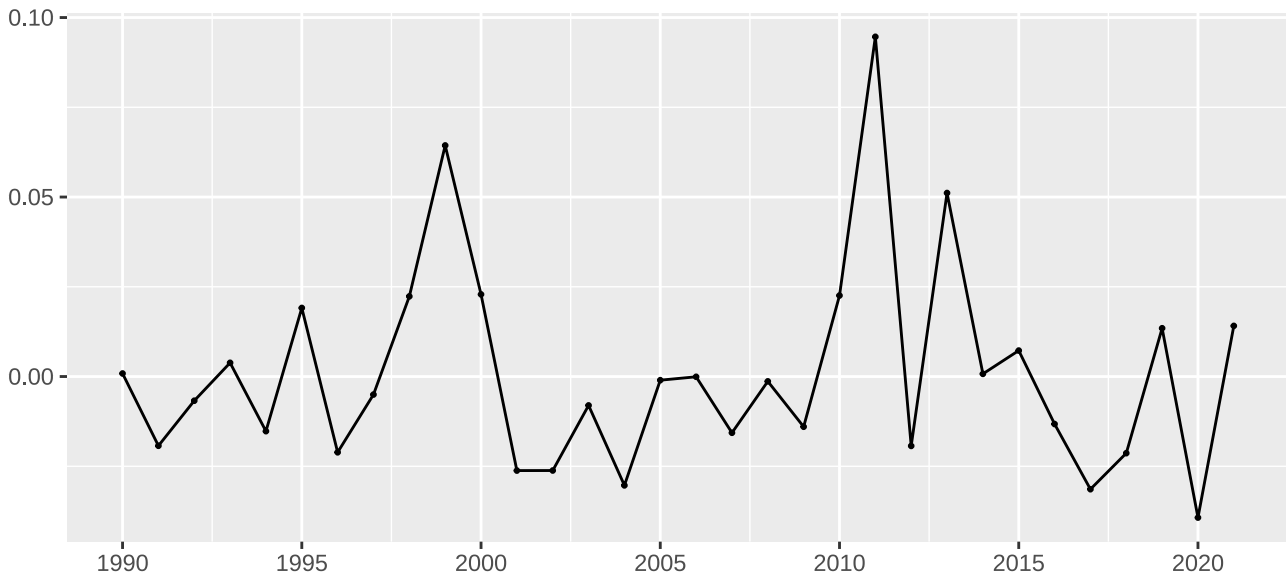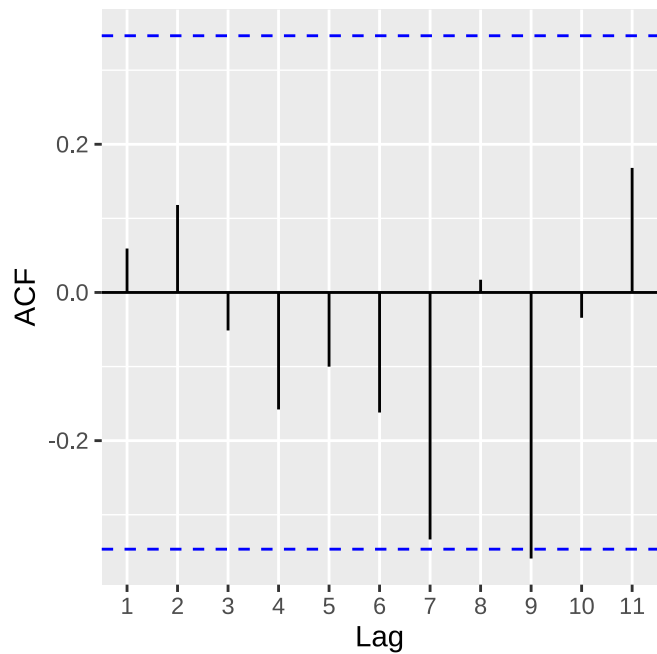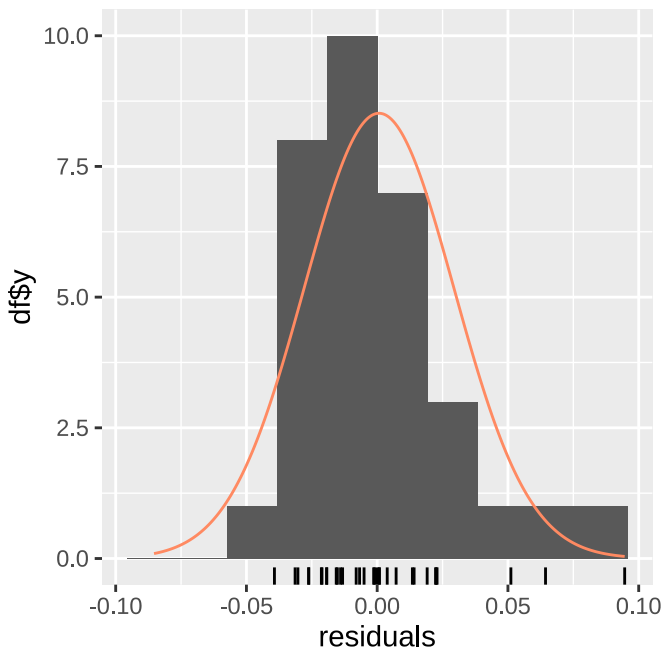

Supplement: Supplementary file 4 [file Data_Sheet_4.pdf]

Residuals from ARIMA(0,2,0)

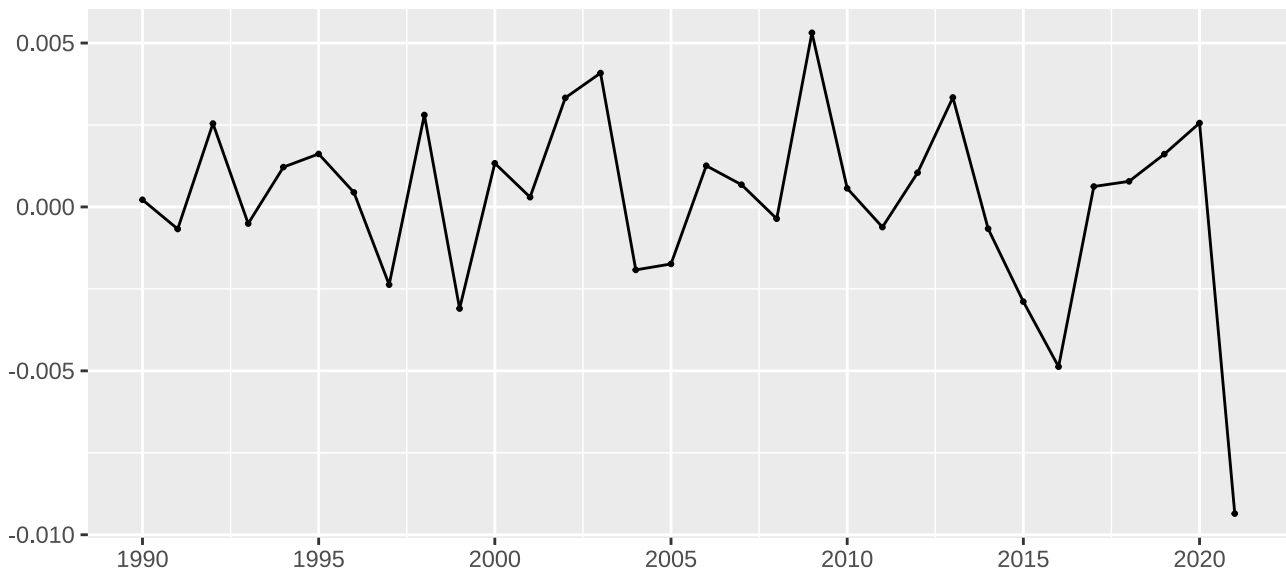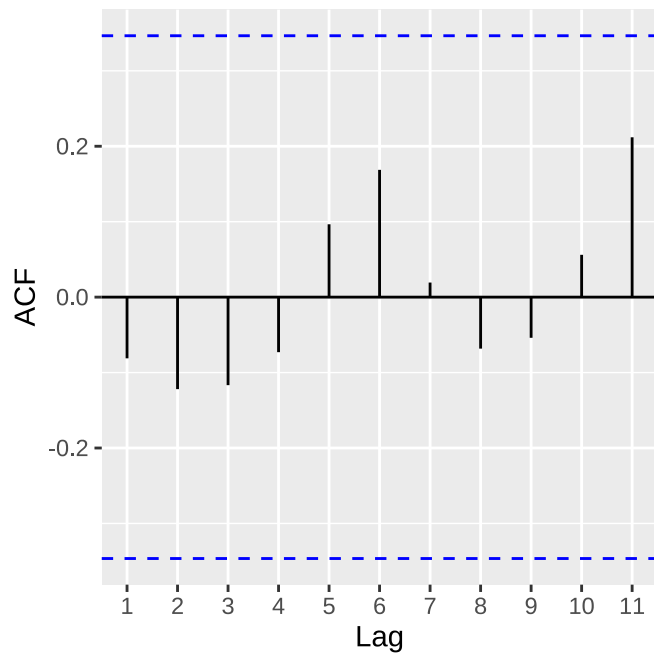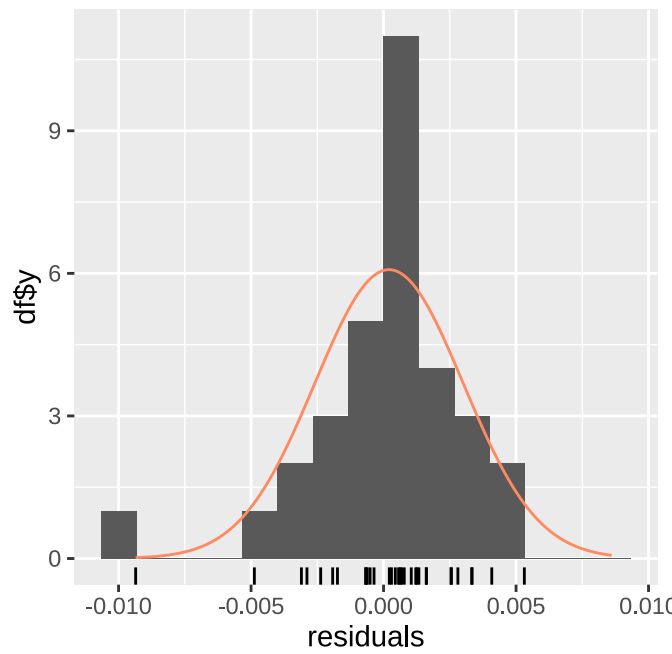

Supplement: Supplementary file 5 [file Data_Sheet_5.pdf]

Residuals from ARIMA(0,2,0)

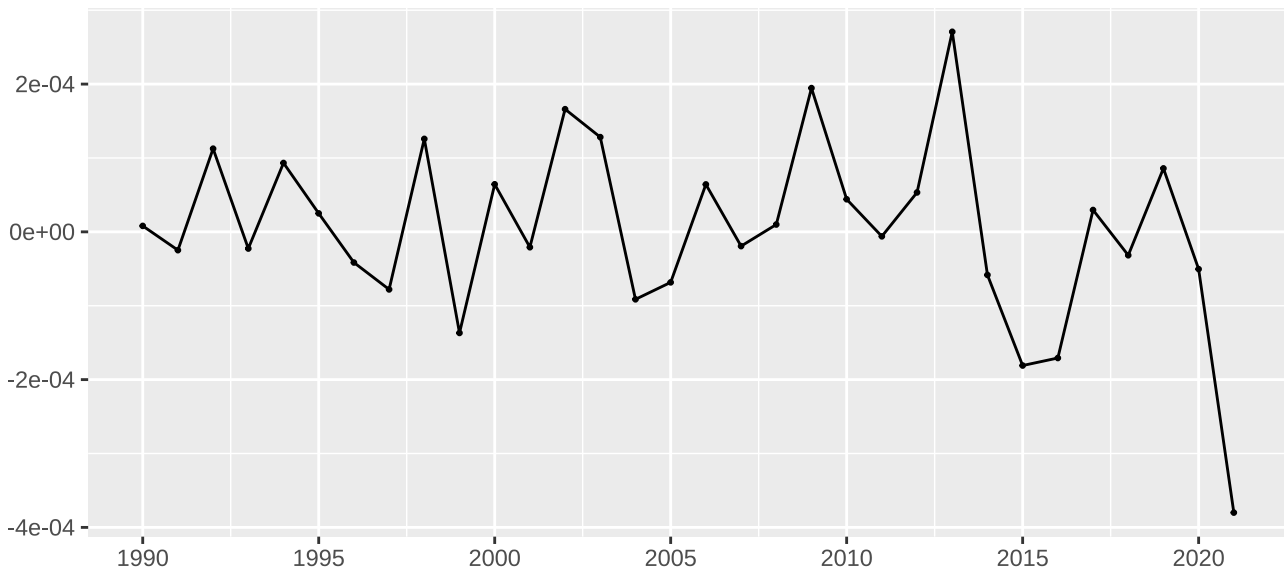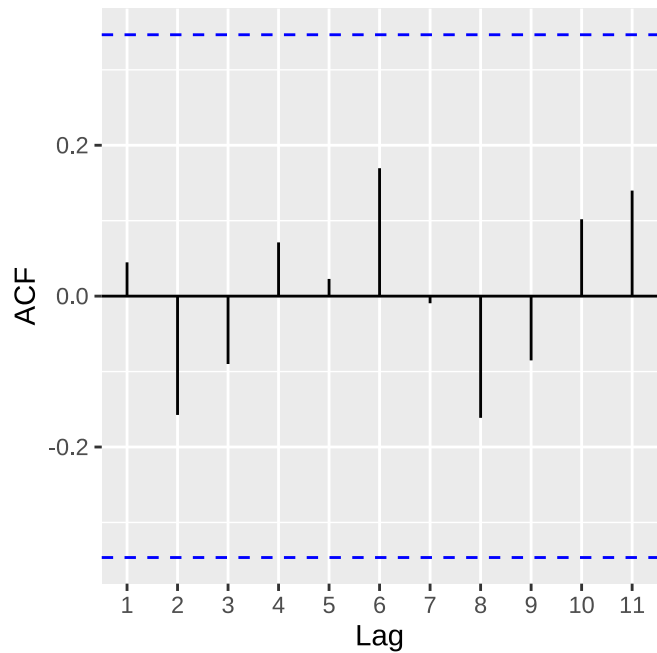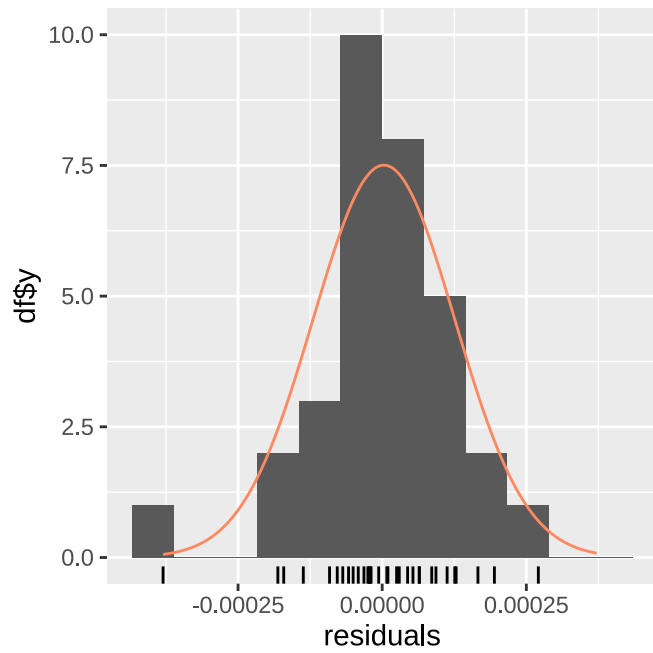

Supplement: Supplementary file 6 [file Data_Sheet_6.pdf]

Residuals from ARIMA(0,2,1)

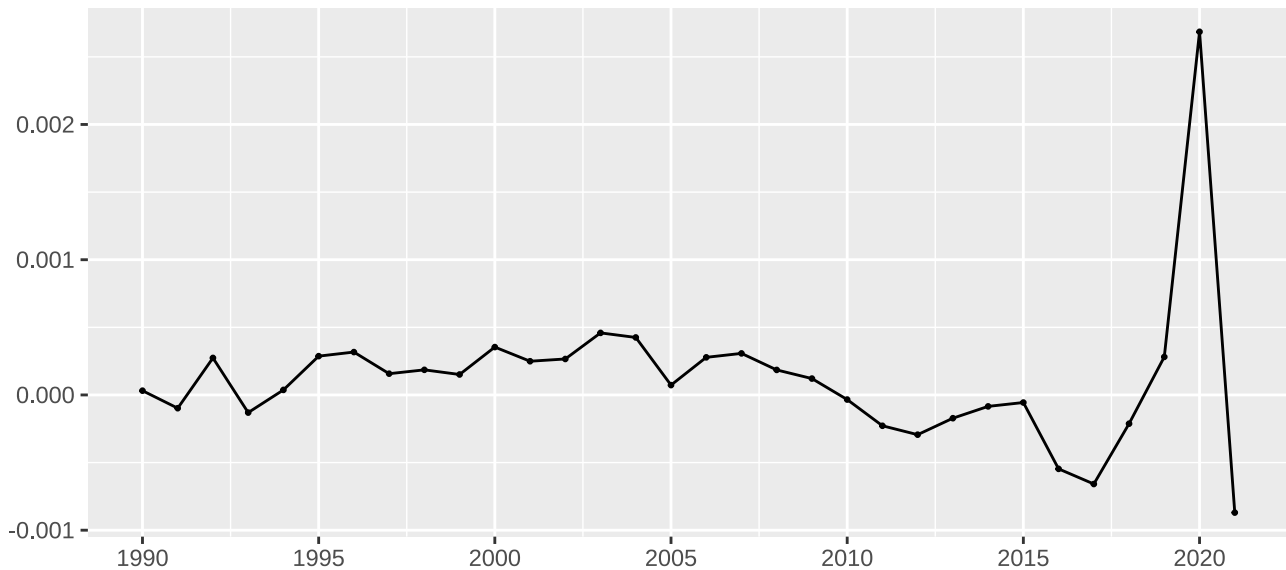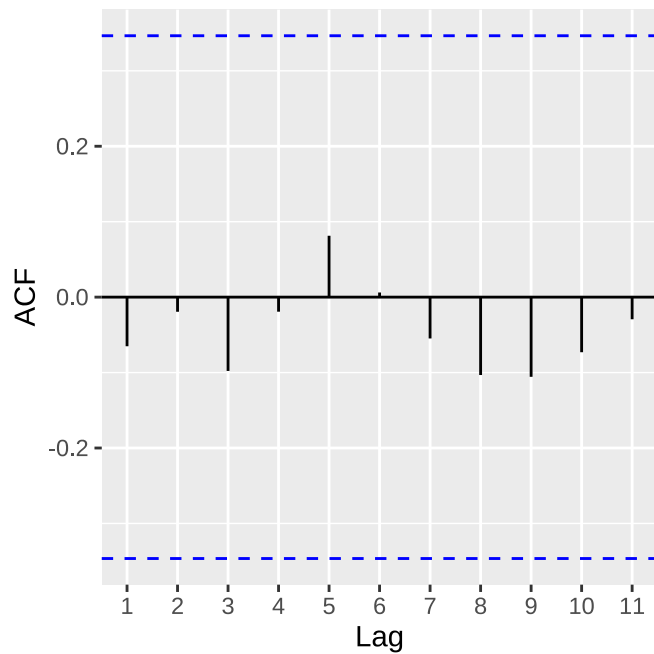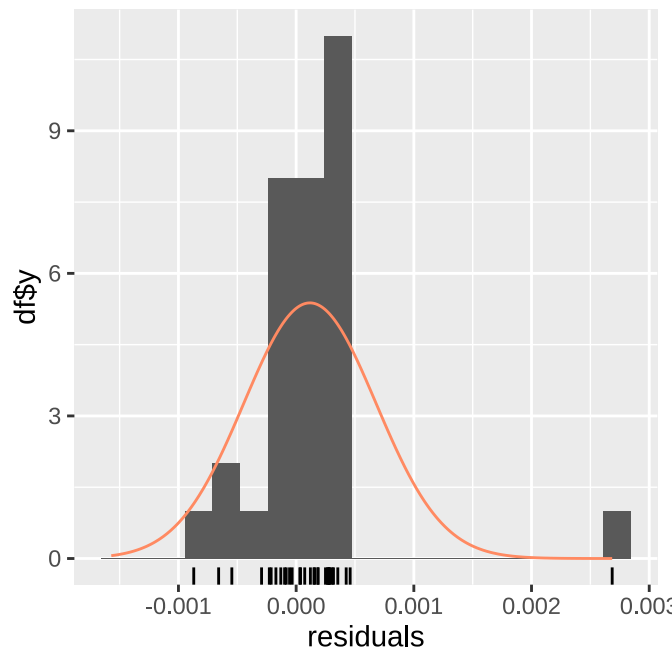

Supplement: Supplementary file 7 [file Data_Sheet_7.pdf]

Residuals from ARIMA(0,2,0)

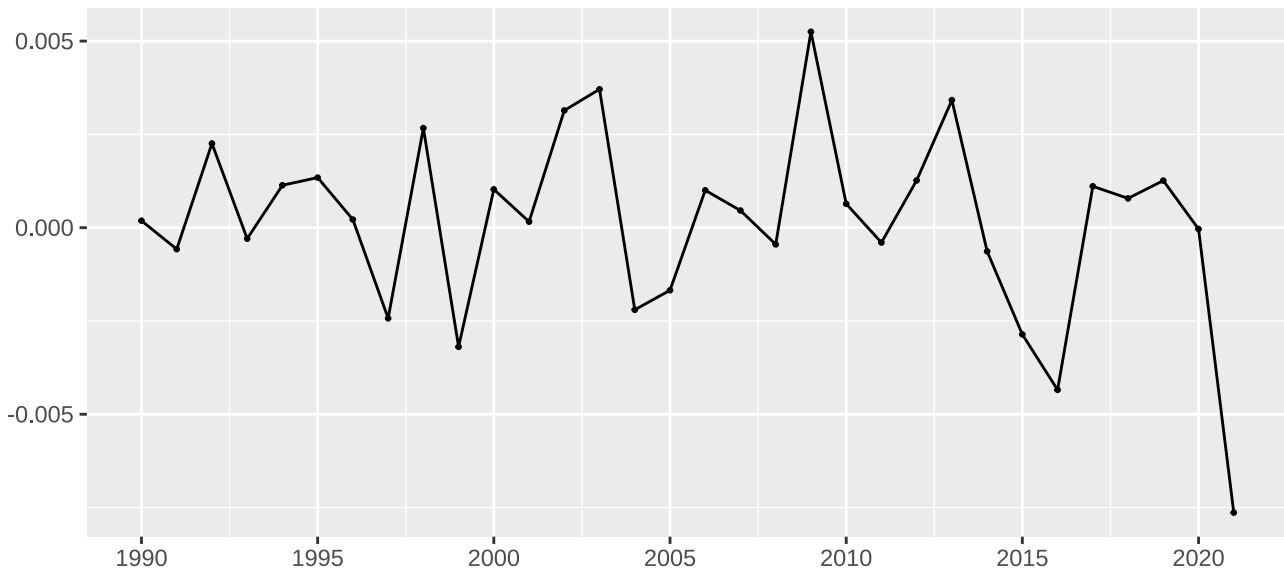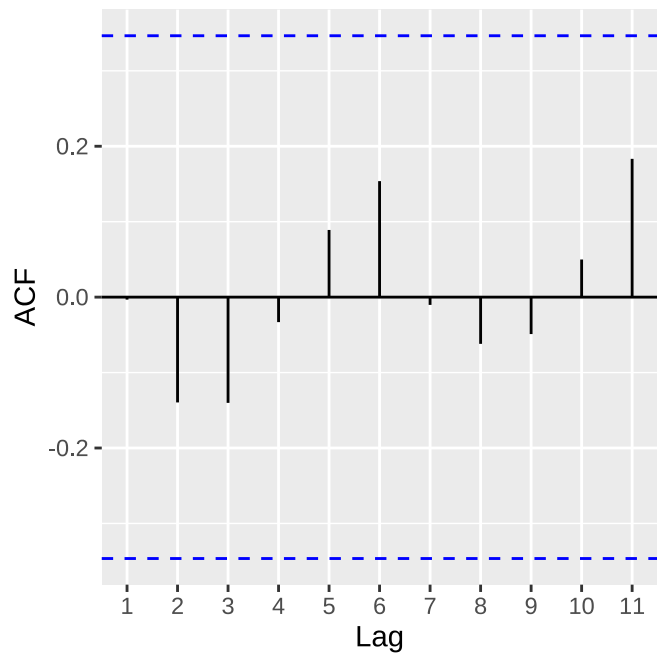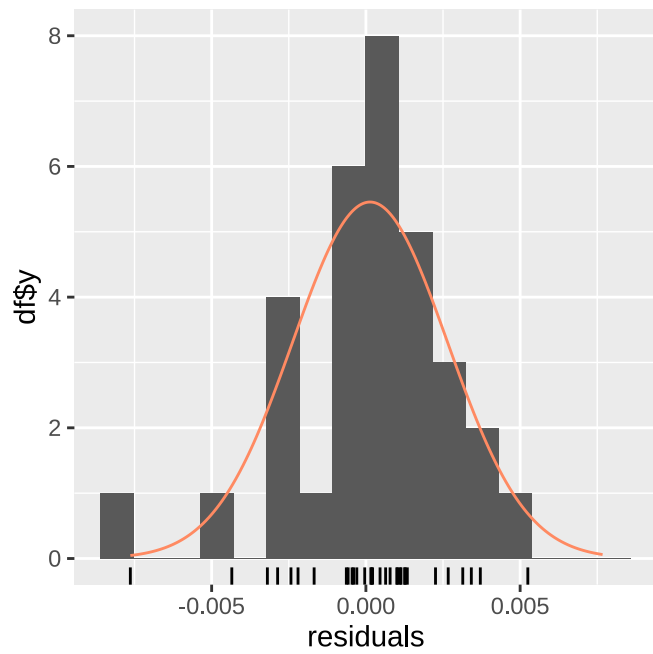

Supplement: Supplementary file 8 [file Data_Sheet_8.pdf]
